# Regulation of drug resistance to enrofloxacin in *Pasteurella multocida* strains from cattle by quorum-sensing acyl-homoserine lactone signaling molecules

**DOI:** 10.3389/fmicb.2026.1766173

**Published:** 2026-01-26

**Authors:** Miao Sun, Zipeng Zhang, Dongxu Han, Xinran Ge, Yongqiang Wang, Jianing Wang, Yue Li, Hengli Chen, Boran Xu, Kexin Ao, Dahan Yang, Kai Liu, Zi Wang

**Affiliations:** 1 College of Animal Science and Technology, Inner Mongolia Minzu University, Tongliao, China; 2 Zhalantun Vocational College, Hulunbuir, China; 3 Tongliao Agriculture and Animal Husbandry Development Center, Tongliao, China; 4 Animal Disease Prevention and Control Center of Horqin District, Tongliao, China; 5 Inner Mongolia Engineering Technology Research Center for Prevention and Control of Beef Cattle Diseases, Tongliao, China; 6 Beef Cattle Industry School of Inner Mongolia Autonomous Region, Tongliao, China

**Keywords:** drug resistance, metabolome sequencing, *Pasteurella multocida*, proteome sequencing, quorum-sensing molecule, transcriptomic sequencing

## Abstract

*Pasteurella multocida* (*Pm*) is a significant cause of respiratory disease in beef cattle, with serious consequences for the cattle industry. The bacterial quorum-sensing (QS) system is used for communication and regulation of various processes, including bacterial growth, virulence, biofilm formation and antimicrobial resistance. This study investigated the effects of the C6 signaling molecule on *Pm* resistance to the antimicrobial enrofloxacin (ENR). Bacteria were divided into groups, with one group (*Pm*-E1) treated with sub-inhibitory concentrations of ENR, a second treated with sub-inhibitory concentrations of ENR + 200 μM C6 (*Pm*-E2), and a control group (*Pm*-YQ). Transcriptomic, proteomic, and metabolomic sequencing of the bacteria was then performed. The results showed that C6 increased the minimum inhibitory concentration of ENR against *Pm* from 0.25 μg/mL to 1 μg/mL. Differentially expressed genes (DEGs), proteins (DEPs), and metabolites (DEMs), with 798 DEGs, 249 DEPs, and 499 DEMs identified in the *Pm*-YQ vs. *Pm*-E1 group, while the *Pm*-E1 vs. *Pm*-E2 group contained 784 DEGs, 301 DEPs, and 397 DEMs. Further analysis of the DEGs, DEPs, and DEMs suggested potential mechanisms by which C6 might induce *Pm* resistance to ENR through regulating the SOS response, CpxAR two-component system, and the ABC transporter system. These findings not only provide insight into the QS-mediated drug resistance mechanisms in *Pm*, but also highlight the potential of targeting the QS system for the development of novel interventions to control pasteurellosis and counteract antimicrobial resistance.

## 1 Introduction

Consumer demand for beef is growing rapidly in China. However, outbreaks of disease in beef cattle have serious effects on the beef industry. One such disease is bovine respiratory disease (BRD), which is common in intensive cattle farming conditions (Pardon and Buczinski, 2020; Myrenås et al., 2024). The development of BRD is associated with multiple factors, including pathogens such as viruses, bacteria, and mycoplasma, as well as predisposing conditions, such as the animal’s health and immune status, stress responses, and the husbandry environment (such as housing and management; Lachowicz-Wolak et al., 2025; Buczinski et al., 2021; Donlon et al., 2023). Among these, bacterial infections represent one of the core pathogenic components, posing significant challenges to health and leading to substantial economic losses (Monteiro et al., 2025). *Pasteurella multocida* (*Pm*), one of the primary opportunistic pathogens responsible for BRD, is frequently found in the upper respiratory tracts of healthy cattle. A subsequent decline in health, increase in stress, or poor husbandry management can induce transformation of the bacterium into a primary or secondary pathogen, leading to disease (Islam et al., 2023; E-Kobon et al., 2017). As a zoonotic pathogen, *Pm* can not only infect the human respiratory tract but may also act as a foodborne pathogen through contamination of beef and milk products. Consumption of undercooked beef or unpasteurized milk may lead to *Pm* infection via the digestive tract, although this appears to be relatively uncommon. Based on differences in its capsular antigen (K antigen), *Pm* can be classified into five serotypes (A, B, D, E, and F). Further subdivision according to lipopolysaccharide antigens (O antigens) yields 16 distinct serotypes (serovars 1–16) that vary in terms of host ranges and disease manifestations (Peng et al., 2019; Yu et al., 2016; Wang et al., 2023; Wang et al., 2021). Current research indicates that all five capsular serotypes can infect cattle, with serotype A primarily causing bovine pneumonia, while serotypes B and E are mainly responsible for hemorrhagic septicemia in cattle and buffalo. There are fewer reports of infections involving serotypes D and F in cattle (Wilson and Ho, 2013; Zhou et al., 2023; Elsayed et al., 2021). It is particularly noteworthy that the A:3 strain is the major serotype implicated in BRD and is often involved in co-infections with other pathogens such as *Mannheimia haemolytica* and *Mycoplasma* species, exacerbating disease severity (Yang et al., 2022).

Currently, antibiotics are the first-line drugs used for the prophylaxis and treatment of *Pm* infections in veterinary practice (Cuevas et al., 2020). Although antibiotics play a crucial role in controlling these infections, excessive and inappropriate use of the drugs has increased selective pressure on the expression of resistance-associated genes, leading to increased bacterial resistance and the emergence and spread of multidrug-resistant strains. The escalating problem of antimicrobial resistance has become a major concern for both veterinarians and livestock producers (Wang et al., 2025). In recent years, fluoroquinolone antibiotics, including ciprofloxacin and enrofloxacin (ENR), have been widely used in China to treat *Pm* infections. However, the widespread and prolonged use of these drugs has led to increased resistance to fluoroquinolone antibiotics in clinical strains (Li et al., 2023).

Quorum sensing (QS) is an important bacterial communication system. This involves the production and release of QS signaling molecules, their binding to specific receptors, and the induction of downstream signaling (Fan et al., 2022). Bacteria produce and sense signaling molecules through the QS mechanism mediated by autoinducers (AIs), enabling mutual information exchange. This type of bacterial communication is controlled by two types of QS systems, known as AI-1 and AI-2 (Pacheco et al., 2021), of which the system utilizing acyl-homoserine lactones (AHLs or AI-1) as signaling molecules in Gram-negative bacteria has been extensively studied (Coquant et al., 2020). The production and recognition of specific signaling molecules enable the coordination of bacterial gene expression and physiological activities. When the concentrations of these signaling molecules and the bacterial population density reach critical thresholds, the bacterial community initiates a series of cascade reactions to modulate the expression of specific genes, enabling bacterial adaptation to environmental changes (Zeng et al., 2023). Studies have found that QS, one of the key regulators of bacterial survival, can regulate a variety of cellular processes, including drug efflux pumps and biofilm formation (Wei and Zhao, 2018; Bäuerle et al., 2018; Wu et al., 2020). Therefore, an understanding of the bacterial QS system offers new directions for the in-depth investigation of the regulatory mechanisms underlying drug resistance, as well as the development of new drugs (Zhao et al., 2020).

In the type I QS system mediated by AHL signaling molecules, LuxI enzymes are responsible for AHL biosynthesis, with derivation of the lactone ring domain from S-adenosylmethionine (SAM). Interaction of AHL with LuxR-type receptors regulates the expression of downstream target genes. However, some bacterial strains cannot synthesize LuxI enzymes themselves but can instead express LuxR or its homologs, and are thus able to respond to exogenous AHL signaling molecules. Previous studies have confirmed that *Escherichia coli*, despite lacking a complete LuxR/LuxI-type QS system, can sense exogenous AHL signals through SdiA, a transcription factor belonging to the LuxR family, and can then regulate the expression of its own drug resistance-related genes (Wang et al., 2025). To date, there have been no investigations of AHL-mediated QS systems in *Pm*. Our preliminary studies demonstrated that exogenous supplementation of AHL signaling molecules significantly influenced drug resistance in *Pm*. However, analyses based on current literature and public genomic databases indicate that no typical LuxR-type receptors or their homologous proteins have been identified in *Pm*. This finding suggests that AHL-mediated regulation of drug resistance in *Pm* may not operate through the canonical QS pathway, but rather involves alternative molecular mechanisms that remain to be elucidated. As the parasitic environment of *Pm* contains a complex variety of strains, making it highly susceptible to interference from exogenous QS signaling molecules, the present study focused on analysis of bovine-derived *Pm*. We explored the effects of exogenous N-octanoyl-L-homoserine lactone (C6), a typical AHL signaling molecule, on enrofloxacin resistance in *Pm*. In addition, combined with transcriptomic, proteomic and metabolomic sequencing, we systematically analyzed the molecular mechanisms underlying C6 regulation of *Pm* resistance. The findings of the study offer a novel perspective for understanding QS-mediated antibiotic resistance in *Pm*, and also provide a theoretical foundation and promising interventional strategies for addressing bacterial antibiotic resistance in clinical practice.

## 2 Materials and methods

### 2.1 Bacterial strain

The capsular serotype A *Pm* was isolated from beef cattle with pneumonia in the Wulagai region of Inner Mongolia and is kept in the Laboratory of Preventive Veterinary Medicine, College of Animal Science and Technology, Inner Mongolia MINZU University (Wang et al., 2024a).

### 2.2 Drug sensitivity testing

The sensitivity of the isolated strains to ENR was determined using the microbroth dilution method recommended by Clinical and Laboratory Standards Institute (CLSI) (CLSI, 2015), with specific procedures performed as described by Wang et al. (2025). ENR (purity≥98%, Yuanye Biotechnology Co. Ltd., Shanghai, China, Product No. S17081) stock solution (2048 μg/mL) was prepared in 1 M NaOH. The experiment was performed with three biological replicates. The MIC was defined as the lowest concentration of the drug at which no visible bacterial growth was observed. The MIC values of ENR for the isolated strains were interpreted and categorized as susceptible (S), intermediate (I), or resistant (R) according to the breakpoint criteria of both CLSI for *Pm* and the National Antimicrobial Resistance Monitoring System (NARMS; Wang et al., 2024b).

### 2.3 Effect of AHL signaling molecules on drug resistance in pm

The L-type molecule N-octanoyl-L-homoserine lactone C6 was dissolved in dimethyl sulfoxide (DMSO) and physiological saline, and the effects of the solvent and C6 on the growth of the bacterial strain were evaluated. The results showed that neither the solvent nor C6 had a significant impact on bacterial growth. As described by Zhang et al., 2018, a preliminary experiment was conducted by adding the AHL C6 at concentrations of 40 μM (2 × 10^−4^ mol/L), 60 μM (3 × 10^−4^ mol/L), 80 μM (4 × 10^−4^ mol/L), 100 μM (5 × 10^−4^ mol/L), 200 μM (1 × 10^−3^ mol/L) and 400 μM (2 × 10^−3^ mol/L) to identify an appropriate concentration for use. Moreover the MIC values were identical for both the 200 μM (1 × 10^−3^ mol/L) and 400 μM (2 × 10^−3^ mol/L) concentrations. Building upon these preliminary findings and relevant literature, 200 μM (1 × 10^−3^ mol/L) was selected for subsequent experiments. This concentration represents the lowest concentration of C6 that showed a maximal effect on the MIC of ENR against *Pm*, thereby minimizing potential non-specific effects associated with higher doses. It is also the classic concentration used in previous studies on the regulation of AHL-mediated resistance in Gram-negative bacteria. The experimental procedure is described in Section 2.2. Bacterial cultures were prepared using BHI medium, and 200 μM (1 × 10^−3^ mol/L) of N-octanoyl-L-homoserine lactone C6 was added to the bacterial suspension containing ENR. The cultures were then incubated at 37 °C for 24 h, after which the MIC values were determined. All experiments were performed with three biological replicates.

### 2.4 Sample preparation

The bacteria were divided into three groups, namely, a group treated with a sub-inhibitory concentration of ENR, a second group treated with the sub-inhibitory ENR concentration plus C6, and a control group. Specifically, Group 1 (*Pm*-E1) was treated with 0.125 μg/mL ENR, Group 2 (*Pm*-E2) was treated with 0.5 μg/mL ENR with the addition of 200 μM (1 × 10^−3^ mol/L) C6, and the control group (*Pm*-YQ) was grown without either ENR or C6. All groups were incubated in BHI medium with shaking at a constant temperature of 37 °C, and the bacteria were harvested at the exponential growth phase (OD_600_ = 0.5).

### 2.5 Transcriptomic sequencing and identification of DEGs

Total RNA was extracted from the *Pm*-YQ, *Pm*-E1, and *Pm*-E2 groups using a Bacterial RNA Extraction Kit (Guangzhou Feiyang Bioengineering Co., Ltd., China) as specified in the manufacturer’s instructions. The RNA samples were sent to Guangzhou Gene Denovo Biotechnology Co. for high-throughput sequencing on an Illumina NovaSeq 6,000 platform (San Diego, CA, United States) and subsequent analysis. The raw reads were filtered to remove low-quality reads, including those containing adapters, nucleotides with quality scores lower than 20, and more than 10% unknown nucleotides, before mapping to the ribosomal RNA (rRNA) database using Bowtie 2 version 2.2.8. The rRNA-mapped reads were removed, and the remaining reads were mapped to the reference genome (accession number CP142011). Genes were annotated using Rockhopper, and expression levels were normalized using the fragments per kilobase of transcript per million mapped reads (FPKM) method. The edgeR package (version 3.12.1) in R^1^ was used for identification of the DEGs using the criteria of fold change ≥ 2 and false discovery rate (FDR) < 0.05. All DEGs were annotated using the Gene Ontology (GO) and the Kyoto Encyclopedia of Genes and Genomes (KEGG) databases.

### 2.6 Real-time quantitative PCR (RT-qPCR)

Eleven DEGs (Supplementary Table S1) were selected for verification of their expression levels using RT-qPCR. Each 20 μL reaction volume contained 10 μL of 2 × SYBR Green Master Mix, 0.4 μL each of the forward and reverse primers (10 μM), 1 μL of template, and 8.2 μL of ddH₂O. The conditions included an initial denaturation at 95 °C for 30 s, followed by 40 cycles of 95 °C for 10 s, and finally 60 °C for 30 s. The 16S rRNA gene was used as an internal control, and relative expression levels were calculated using the 2^-∆∆Ct^ method.

### 2.7 Metabolomic sequencing and identification of DEMs

Bacterial samples prepared as described in Section 2.4 were sent to Shanghai Personalbio Biotechnology Co., Ltd. for metabolomic analysis. Chromatographic analysis was performed using an ACQUITY UPLC HSS T3 column (100 Å, 1.8 μm, 2.1 × 100 mm). An Orbitrap Exploris 120 mass spectrometer (Thermo Fisher Scientific, Waltham, MA, United States) was used to collect DDA mass spectrometry data in both positive and negative ion modes under the control of Xcalibur software (version 4.7, Thermo Fisher Scientific). Compound Discoverer 3.3 (version 3.3.2.31, Thermo Fisher Scientific) was used for peak extraction, alignment, correction, and other operations on the offline data. Peaks that were not detected in at least 50% of the QC samples were removed, while the missing values of undetected peaks were filled using Fill Gaps software, and the summed total peak area was normalized. *p*-values were calculated using statistical tests, the variable importance in projection (VIP) was determined by the OPLS-DA dimension reduction method, and the fold change was assessed to determine differences between two groups. Metabolites with *p*-values < 0.05 and VIP > 1 were considered statistically significant. Clustering analysis of the abundance of DEMs was performed using the pheatmap package (version 1.0.12) in R. Correlation analysis of DEMs was conducted using corrplot (version 4.0.3). Functional analysis of DEMs was undertaken by KEGG enrichment analysis using clusterProfiler (version 4.6.0), revealing significantly enriched metabolic pathways.

### 2.8 Proteomic sequencing and identification of DEPs

Bacterial samples prepared as described in Section 2.4 were sent to Shanghai Personalbio Biotechnology Co., Ltd. for protein extraction and proteomic analysis. Samples were separated using the Vanquish Neo ultra-high-performance liquid chromatography (UHPLC) system. Data-independent acquisition (DIA) analysis was undertaken with a Vanquish Neo system (Thermo Fisher Scientific) for chromatographic separation. Samples separated by nano-flow high-performance liquid chromatography underwent DIA mass spectrometry analysis using the Orbitrap Astral high-resolution mass spectrometer (Thermo Fisher Scientific). The raw mass spectrometry (MS) data were analyzed using DIA-NN (v1.8.1) with a library-free method. All data were based on 99% protein identification confidence with FDR ≤ 1%. Protein intensities were normalized and preliminarily filtered using MaxLFQ, followed by imputation of missing values using the KNN method. DEPs with fold change > 2 and *p* < 0.05 were identified using t-tests. Functional enrichment analyses were performed using the R package clusterProfiler (v4.10.0) for GO and KEGG analyses.

### 2.9 Statistical analysis

Statistical analysis was performed using GraphPad Prism 5 (Graph Software, San Diego, CA, United States). All data are expressed as the mean ± standard error based on three independent experiments. A *p* < 0.05 was considered statistically significant.

## 3 Results

### 3.1 Drug sensitivity results

The MIC value of ENR against *Pm*-YQ was determined to be 0.25 μg/mL. Treatment with 200 μM (1 × 10^−3^ mol/L) C6 resulted in a four-fold increase in the MIC of ENR against this strain, increasing from 0.25 to 1 μg/mL.

### 3.2 DEG analysis

#### 3.2.1 Identification of DEGs

DEGs were identified in edgeR using the criteria |log2FC| > 1 and FDR < 0.05. A total of 798 DEGs were found in the *Pm*-YQ vs. *Pm*-E1 comparison group (*p* < 0.05), of which 294 genes were up-regulated and 504 were down-regulated. As shown in Table 1, the most significantly up-regulated DEGs were *dsbA*, *rpoH*, *trmA*, *htpX*, *dnaK*, *clpB,* and *hslV*, while the most significantly down-regulated DEGs were *bioD*, *glpQ*, *glpK*, *adhE*, *fumC*, *frdC,* and *frdD*. The *Pm*-E1 vs. *Pm*-E2 yielded 784 DEGs (*p* < 0.05), including 590 up-regulated and 194 down-regulated genes. As shown in Table 2, the most significantly up-regulated genes were *rapA*, *recC*, *mlaC*, *dnaE*, *brnQ*, *fabG,* and *pnuC*, while the most significantly down-regulated genes were *mglB*, *galT*, *aspA*, *mglA*, *dhaL*, *rpiB,* and *galK*. Volcano plots (Supplementary Figure S1) and heatmaps (Supplementary Figure S2) illustrate the identified DEGs.

**Table 1:** Most significant DEGs identified in the *Pm*-YQ vs. *Pm*-E1 group.

ID	*Pm*-YQ_FPKM	*Pm*-E1_FPKM	Log_2_(FC)	Symbol
VA608_RS07220	149.4423333	1396.290667	3.223938508	*dsbA*
VA608_RS08785	1492.384333	13422.17067	3.168926981	*rpoH*
VA608_RS07210	106.7783333	1425.115333	3.738387839	*trmA*
VA608_RS02180	3221.214333	28610.85133	3.150885863	*htpX*
VA608_RS03540	2787.398333	23341.481	3.065905011	*dnaK*
VA608_RS09895	1425.731	11224.47133	2.976873785	*clpB*
VA608_RS07455	1205.862	9187.122667	2.929548278	*hslV*
VA608_RS03040	1166.327333	40.55466667	−4.845960996	*bioD*
VA608_RS08030	1267.226333	51.24633333	−4.628081627	*glpQ*
VA608_RS08040	1466.220333	66.8	−4.456110005	*glpK*
VA608_RS08080	1410.913667	79.977	−4.140900737	*adhE*
VA608_RS04035	1633.276	299.805	−4.030634758	*fumC*
VA608_RS05155	2243.897333	138.834667	−4.014566913	*frdC*
VA608_RS05160	6554.273333	433.486	−3.918378533	*frdD*

**Table 2:** Most significant DEGs identified in the *Pm*-E1 vs. *Pm*-E2 group.

ID	*Pm*-E1_FPKM	*Pm*-E2_FPKM	Log_2_(FC)	Symbol
VA608_RS07030	43.173	262.1566667	2.602227983	*rapA*
VA608_RS01035	119.1963333	615.6833333	2.368848658	*recC*
VA608_RS05265	318.07	1522.838333	2.259346581	*mlaC*
VA608_RS06030	119.3943333	543.0636667	2.185386979	*dnaE*
VA608_RS06860	183.837	821.3893333	2.159639052	*brnQ*
VA608_RS06645	230.5836667	986.3196667	2.096764977	*fabG*
VA608_RS07025	563.9193333	2334.162	2.049343983	*pnuC*
VA608_RS00610	4380.217333	44.55233333	−6.619357652	*mglB*
VA608_RS00620	1114.437	111.538	−4.905670351	*galT*
VA608_RS00265	3867.065333	135.903	−4.830589930	*aspA*
VA608_RS00605	464.826	19.32433333	−4.588200219	*mglA*
VA608_RS10180	1776.514667	87.58533333	−4.342216487	*dhaL*
VA608_RS10195	967.877	63.372	−3.932906266	*rpiB*
VA608_RS00625	603.25	44.065	−3.775050901	*galK*

#### 3.2.2 GO functional annotation of DEGs

**Figure 1:**
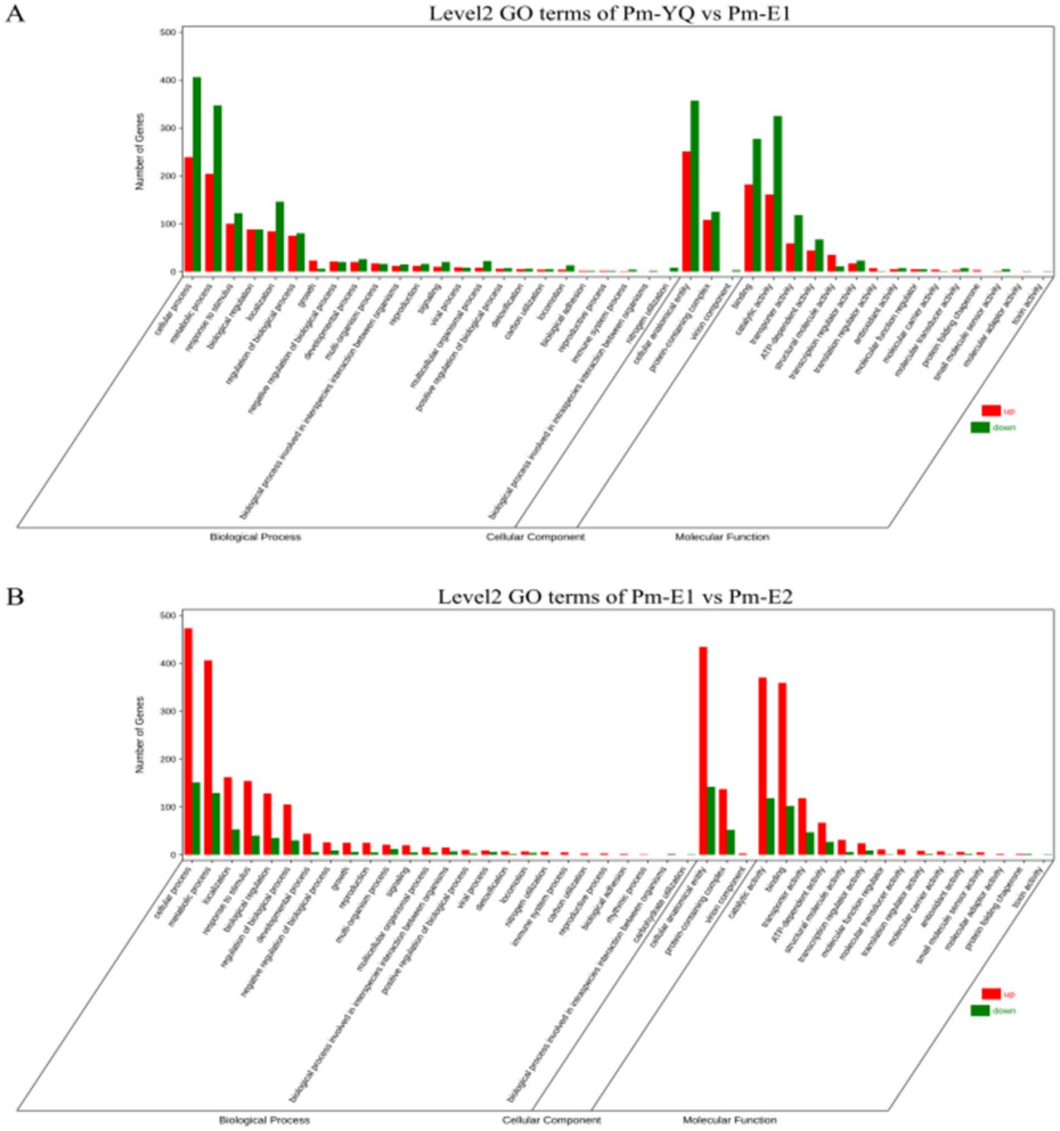
GO functional annotation of DEGs. **(A)** Functional annotation of *Pm*-YQ vs. *Pm*-E1 GO DEGs. **(B)** Functional annotation of *Pm*-E1 vs. *Pm*-E2 GO DEGs.

To compare the functions of the DEGs between the different groups, the up- and down-regulated DGEs identified via edgeR were analyzed using GO, with a threshold value *p* < 0.05. The GO categories were cellular component (CC), molecular function (MF), and biological process (BP). The DEGs in the *Pm*-YQ vs. *Pm*-E1 group were classified into 15 MFs, 3 CCs, and 24 BPs, while those in the *Pm*-E1 vs. *Pm*-E2 comparison included 15 MFs, 3 CCs, and 26 BPs (Figures 1A,B). Classification of the GO functional annotation results in Level 2 GO terms showed that most DEGs were significantly enriched in metabolic processes, cellular processes, response to stimulus, and localization in the BP category, cellular anatomical entity and protein-containing complex in the CC category, and transporter activity, catalytic activity, and binding in MFs.

#### 3.2.3 KEGG enrichment analysis of DEGs

**Figure 2:**
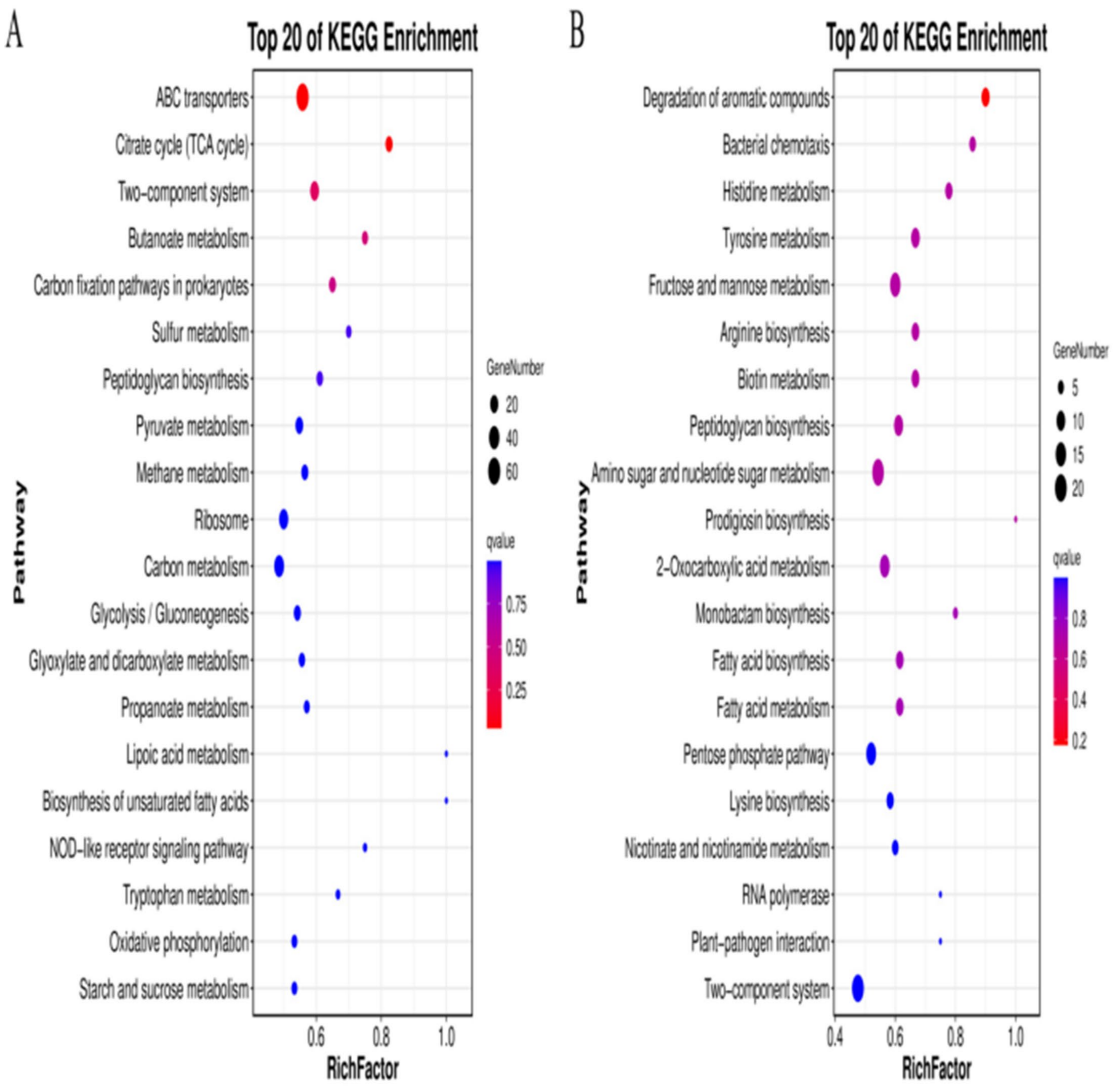
KEGG enrichment analysis of DEGs. **(A)** *Pm*-YQ vs. *Pm*-E1 DEGs. **(B)** *Pm*-E1 vs. *Pm*-E2 DEGs.

To investigate the pathways influencing drug resistance in *Pm*, the DEGs were further evaluated using the KEGG database. The results are shown in Figure 2, with higher RichFactors indicating higher degrees of enrichment. The number of DEGs in the individual pathways is indicated by the size of the dots, with larger dots representing greater numbers; dot colors indicate the q-value corrected by multiple hypothesis testing, with values ranging from 0 to 1, and the smaller the value, the more significant the enrichment. The results showed that the DEGs were primarily enriched in pathways related to metabolism, biochemical processes, and signal transduction, as well as in several specific pathways, including ATP-binding cassette (ABC) transporters, the tricarboxylic acid (TCA) cycle, two-component systems, ribosomes, RNA polymerases, and bacterial chemotaxis. To verify the reliability of the transcriptomic result, 11 DEGs (*recX*, *recA*, *LexA*, *Lon*, *rpoC*, *dnaE*, *mglB*, *otnI*, *glpT*, *glpA,* and *nrdD*) were randomly selected for RT-qPCR verification. Measurement of the expression levels of these 11 genes showed that their expression was consistent with the transcriptome sequencing results (Supplementary Figures S3A–D), indicating the reliability of the transcriptome sequencing results.

### 3.3 DEM analysis

#### 3.3.1 Identification of DEMs

DEMs were identified using a combination of univariate and multivariate statistical analyses, using the criteria of VIP > 1 and *p*-value < 0.05. The *Pm*-YQ vs. *Pm*-E1 comparison identified 499 DEMs (*p* < 0.05), including 203 up accumulated metabolites and 296 down accumulated metabolites. The *Pm*-E1 vs. *Pm*-E2 group contained 397 DEMs (*p* < 0.05), of which 182 were up accumulated metabolites and 215 down accumulated metabolites. Tables 3, 4 show a selection of up accumulated and down accumulated DEMs in *Pm*-YQ vs. *Pm*-E1 and *Pm*-E1 vs. *Pm*-E2 groups, respectively. Volcano plots (Supplementary Figure S4) and heatmaps (Supplementary Figure S5) illustrate the identified DEMs.

**Table 3:** DEMs in the *Pm*-YQ vs. *Pm*-E1 group.

Index	*Pm*-E1	*Pm*-YQ	Log_2_(FC)	Compound
ME0112917	22299334.89	598509.6083	5.219482462	DL-Glyceraldehyde 3-phosphate
MEDN1714	245825655.6	9065391.543	4.761122347	Isokaurenoic acid
MEDP1658	241500871.3	10308544.56	4.550115833	LPC(O-18:1)
ME0103590	68961967.87	3309714.177	4.381022405	Guanosine-5′-monophosphate
ME0006430	28866070.47	1837285.967	3.973726628	Buprenorphine
ME0118870	949413.015	25302070.97	−4.736075837	2-Pyridinemethanethiol
ME0103527	1354337.723	34671506.80	−4.678091091	Dephospho coenzyme a
ME0004104	735115.7883	15656810.53	−4.412675032	3-methoxybenzene-1,2-diol
ME0055436	1268035.73	23067707.92	−4.185207357	Stearaldehyde
ME0154890	784254.1267	13250897.41	−4.078625042	Pantetheine

**Table 4:** DEMs in the *Pm*-E1 vs. *Pm*-E2 group.

Index	*Pm*-E2	*Pm*-E1	Log_2_(FC)	Compound
ME0127456	42475368.27	21551.42333	10.94462759	Dimethyl sulfoxide
ME0008586	1382314.877	13998.13667	6.625707675	Dimethylglycine
ME0142285	30282384.54	401356.56	6.237450524	2-Chloroethanol
ME0106116	11252712.96	328391.48	5.098712361	Hydrogen carbonate
ME0109743	18729405.19	716738.0333	4.707715361	Sodium fluoroacetate
ME0126585	16824636.21	3,133,371,126	−7.540996537	Unii-yxj027jnd6
ME0111541	41689912.62	2,104,327,381	−5.657517011	Stearoylglycerone phosphate
ME0061660	235370.6317	7358493.758	−4.966404261	Phytol
ME0015313	94798224.25	2,176,413,140	−4.520948599	8-Geranylumbelliferone
ME0052562	518944141.6	6,221,840,623	−3.583690277	Epiandrosterone

#### 3.3.2 KEGG enrichment analysis of DEMs

**Figure 3:**
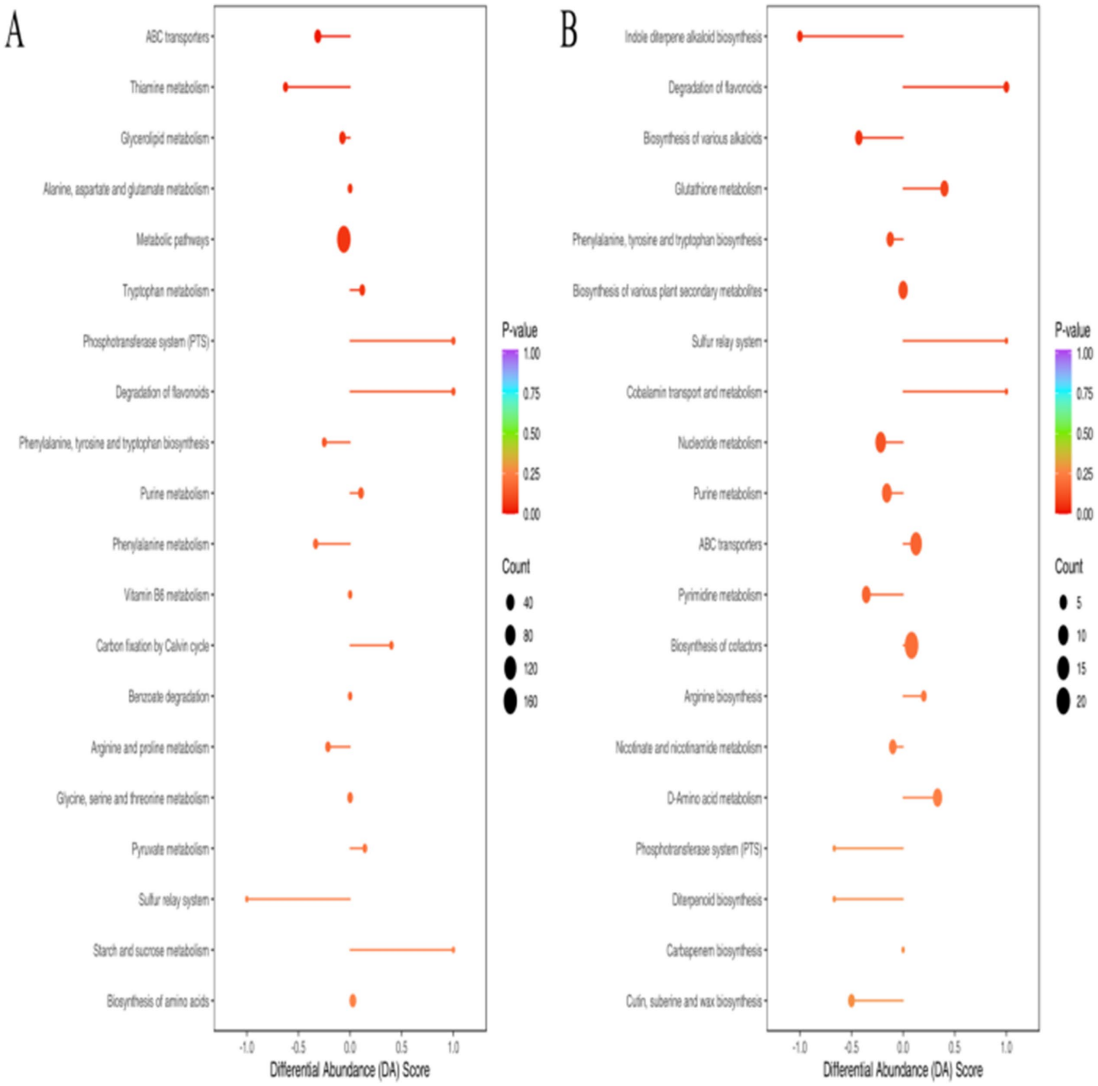
KEGG enrichment analysis of DEMs. **(A)** *Pm*-YQ vs. *Pm*-E1 group. **(B)** *Pm*-E1 vs. *Pm*-E2 group.

The results of the KEGG enrichment analysis revealed that the DEMs were primarily enriched in pathways involved in the synthesis, metabolism, and transport of various substances. Several of these pathways are associated with bacterial resistance, including pathways involving ABC transporters, nucleotide metabolism, purine metabolism, and pyrimidine metabolism (Figure 3). In the *Pm*-YQ vs. *Pm*-E1 group, DEMs enriched in ABC transporters and general metabolic pathways were significantly down-regulated, whereas those associated with purine metabolism were up-regulated. Conversely, in the *Pm*-E1 vs. *Pm*-E2 group, DEMs enriched in nucleotide metabolism, purine metabolism, and pyrimidine metabolism were significantly down-regulated, while up-regulation was observed for DEMs enriched in ABC transporters.

### 3.4 Analysis of DEPs

#### 3.4.1 Identification of DEPs

DEPs were identified using t-tests with the criteria of fold change ≥ 1.5 or fold change ≤0.6667 and *p*-value < 0.05. A total of 249 DEPs (*p* < 0.05) were identified in the *Pm*-YQ vs. *Pm*-E1 group, of which 103 were up-regulated and 146 were down-regulated. In the *Pm*-E1 vs. *Pm*-E2 group, 301 DEPs (*p* < 0.05) were detected, including 152 up-regulated and 149 down-regulated proteins. Representative up- and down-regulated DEPs from the *Pm*-YQ vs. *Pm*-E1 and *Pm*-E1 vs. *Pm*-E2 comparisons are presented in Tables 5 and 6, respectively. Volcano plots (Supplementary Figure S6) and heatmaps (Supplementary Figure S7) illustrate the identified DEPs.

**Table 5:** DEPs in the *Pm*-YQ vs. *Pm*-E1 group.

Index	*Pm*-E1	*Pm*-YQ	Log_2_(FC)	Protein
Q9CNU3	2.005298719	0.19065133	10.51814702	recN
Q9CND0	3.254807165	0.410771844	7.923637445	PM0502
Q9CKE1	0.123164356	0.016185196	7.609692012	PM1681
P95526	25.48503758	4.878760779	5.223670257	recA
Q9CKG8	1.320270664	0.254050646	5.196879771	PM1650
Q9CLG8	0	0.030813855	0	PM1266
Q9CPL2	0	0.015458207	0	nrfc
Q9CK78	0.005147893	0.256121372	0.020099428	dmsB
Q9CN08	0.057338235	0.769718084	0.074492514	bioD1
Q9CK75	0.005281714	0.069151066	0.076379365	PM1758

**Table 6:** DEPs in the *Pm*-E1 vs. *Pm*-E2 group.

Index	*Pm*-E1	*Pm*-E2	Log_2_(FC)	Protein
Q9CK78	0.005147893	0.160782646	31.23270954	dmsB
Q9CLK3	0.001995904	0.024553905	12.30214904	comE
Q9CKZ7	0.987482497	11.28150885	11.42451525	adh2
Q9CKB9	0.244574259	2.507669827	10.25320421	trx
Q9CK75	0.005281714	0.038409233	7.272114644	PM1758
P57845	1.396264261	0.82763695	0.592750937	minC
P57858	0.635828642	0.38585652	0.606856146	pyrD
P57881	6.308586154	4.185998772	0.663539923	serC
P57884	0.110931011	0.057778115	0.520847286	metXA
P57899	0.425342084	0.272431036	0.640498663	galK

#### 3.4.2 GO functional annotation and enrichment analysis of DEPs

**Figure 4:**
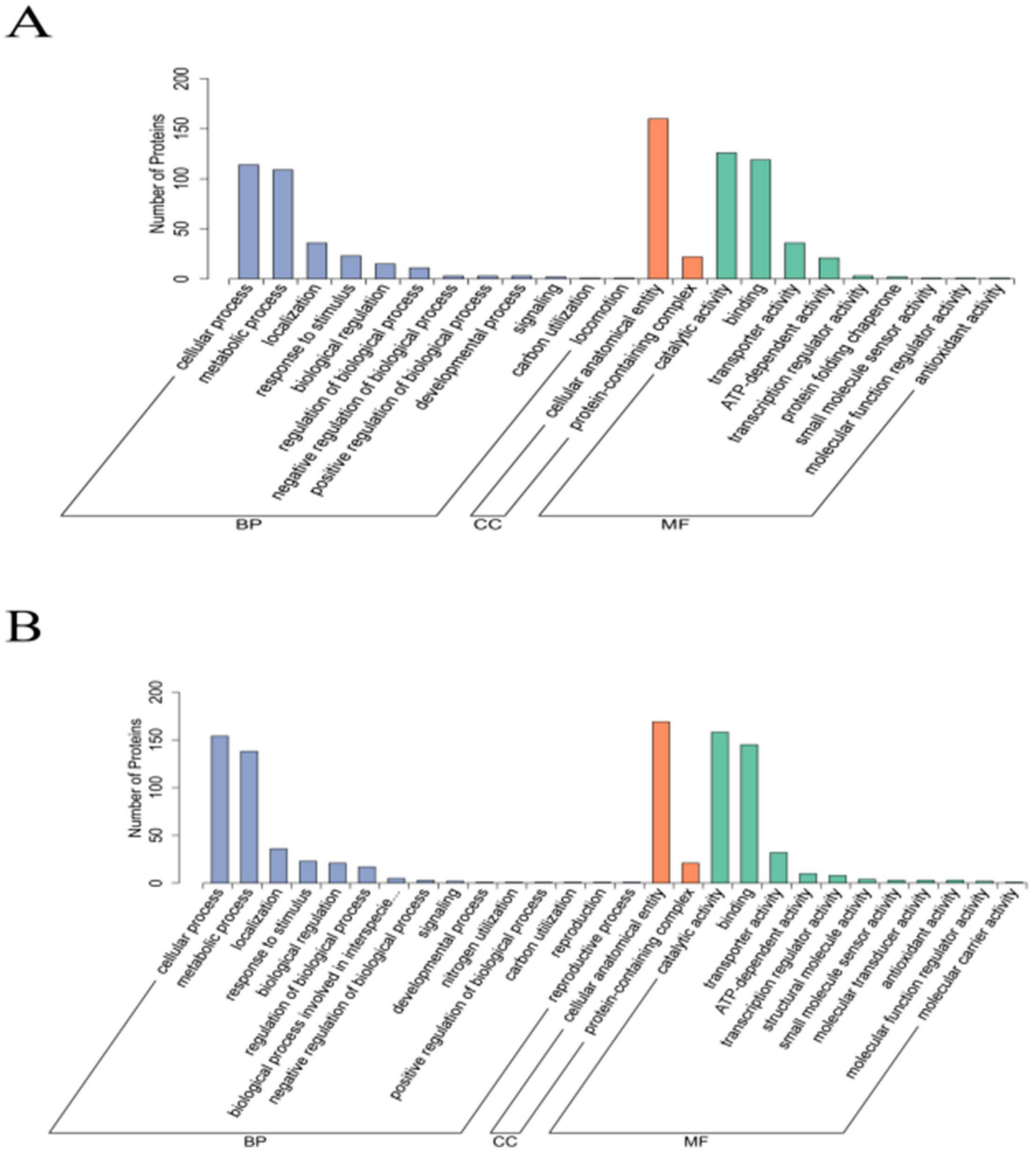
GO functional annotation of DEPs. **(A)** GO functional annotation of *Pm*-YQ vs. *Pm*-E1 DEPs. (B) GO functional annotation of *Pm*-E1 vs. *Pm*-E2 DEPs.

**Figure 5:**
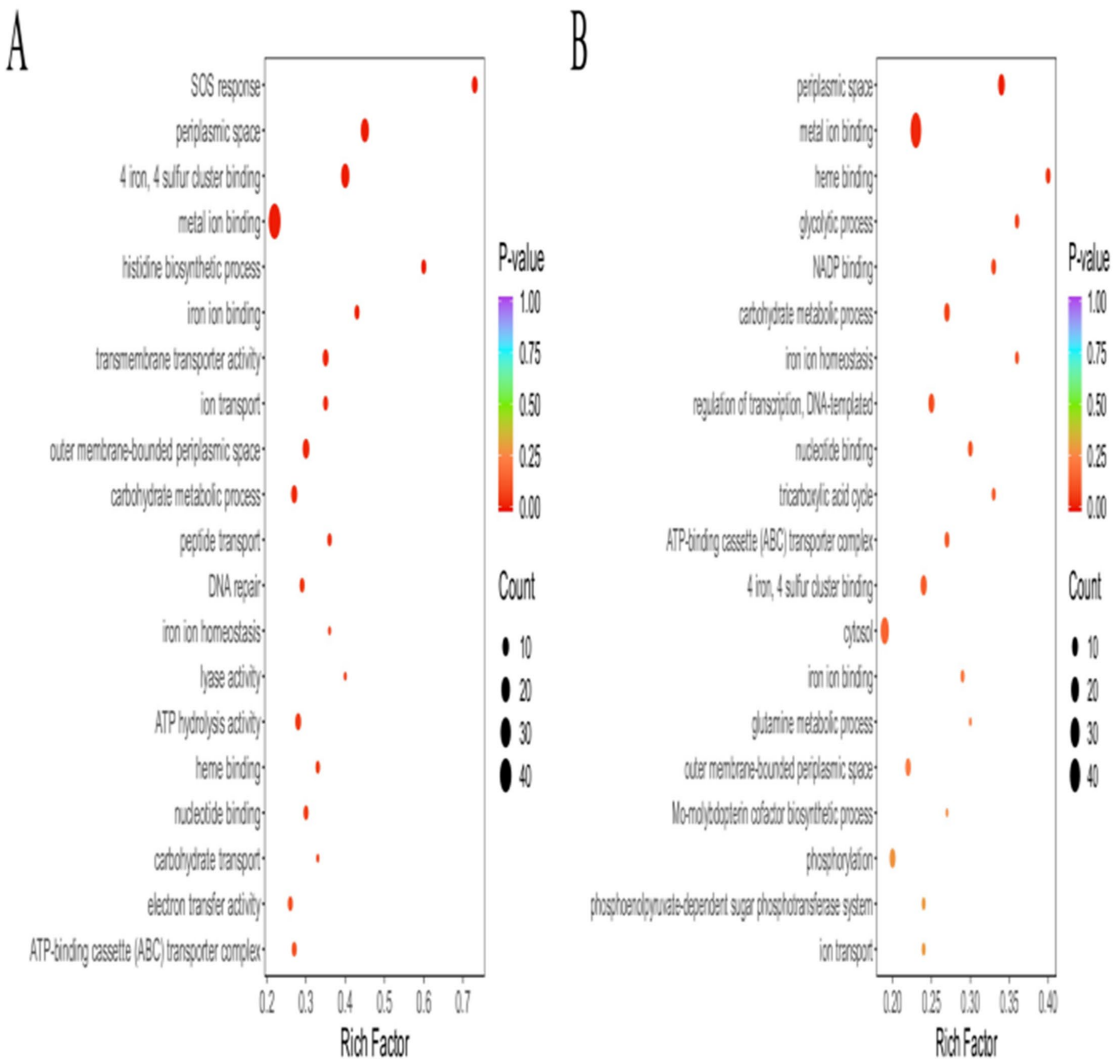
GO enrichment analysis of DEPs. **(A)** GO enrichment analysis of *Pm*-YQ vs. *Pm*-E1 DEPs. **(B)** GO enrichment analysis of *Pm*-E1 vs. *Pm*-E2 DEPs.

The DEPs identified in the *Pm*-YQ vs. *Pm*-E1 group were found to be classified into 9 MFs, 2 CCs, and 11 BPs, while those in the *Pm*-E1 vs. *Pm*-E2 comparison included 11 MFs, 2 CCs, and 15 BPs (Figure 4). Classification of the GO functional annotation results in Level 2 GO terms showed that most of the DEGs were significantly enriched in metabolic processes, cellular processes, response to stimulus, and localization in the BP category, cellular anatomical entity and protein-containing complex in the CC category, and transporter activity, catalytic activity, and binding in MFs. The GO enrichment results indicated that the DEPs were significantly enriched in terms related to various damage repair processes and transport functions, including the SOS response, DNA repair, ABC transporter complex, and transmembrane transporter activity (Figure 5).

#### 3.4.3 KEGG enrichment analysis of DEPs

**Figure 6:**
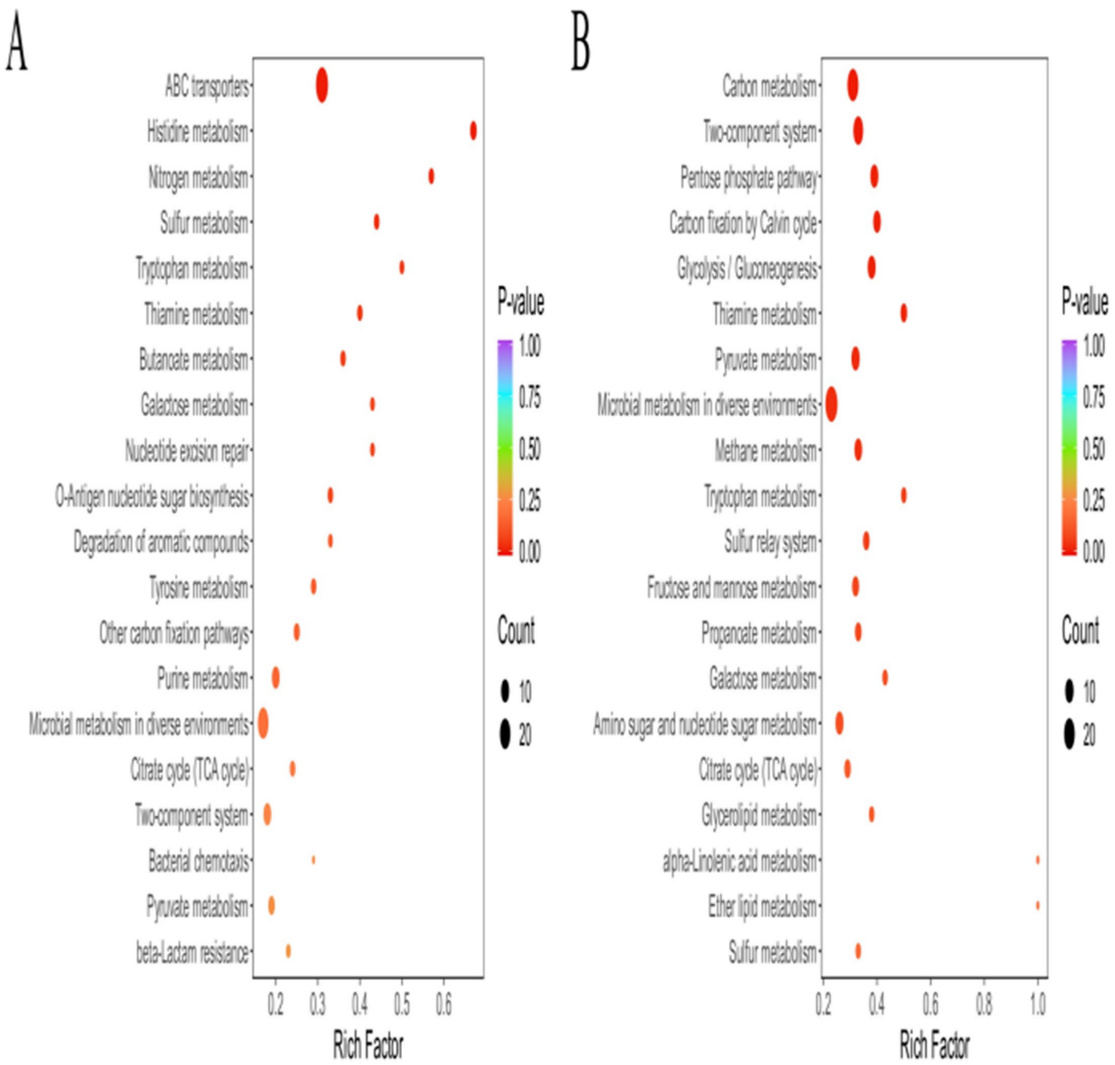
KEGG enrichment analysis of DEPs. **(A)** *Pm*-YQ vs. *Pm*-E1 group of DEPs. **(B)** *Pm*-E1 vs. *Pm*-E2 group of DEPs.

KEGG enrichment analysis showed that in the *Pm*-YQ vs. *Pm*-E1 comparison, DEPs were enriched not only in various metabolic pathways but also in specific pathways, such as ABC transporters, nucleotide excision repair, two-component systems, and bacterial chemotaxis. In contrast, DEPs from the *Pm*-E1 vs. *Pm*-E2 comparison were primarily enriched in biosynthetic, metabolic and two-component system pathways, including carbon metabolism, pentose phosphate pathway, and microbial metabolism in different environments (Figure 6).

### 3.5 Combined transcriptomic, metabolomic, and proteomic analyses

#### 3.5.1 Combined KEGG enrichment analysis

**Figure 7:**
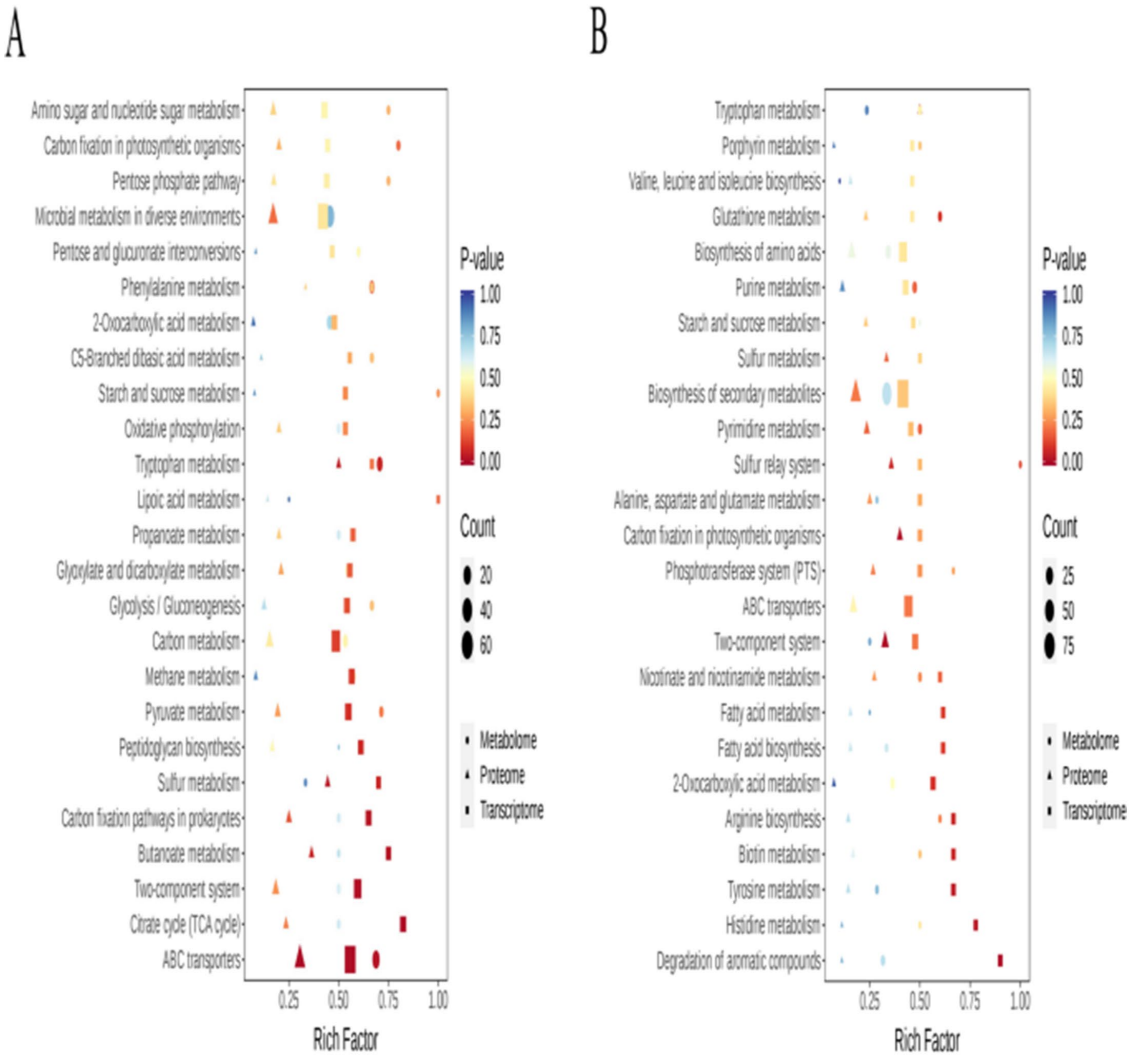
Combined KEGG enrichment analysis of data from the three omics analyses: **(A)** KEGG enrichment analysis of *Pm*-YQ vs. *Pm*-E1; **(B)** KEGG enrichment analysis of *Pm*-E1 vs. *Pm*-E2.

**Figure 8:**
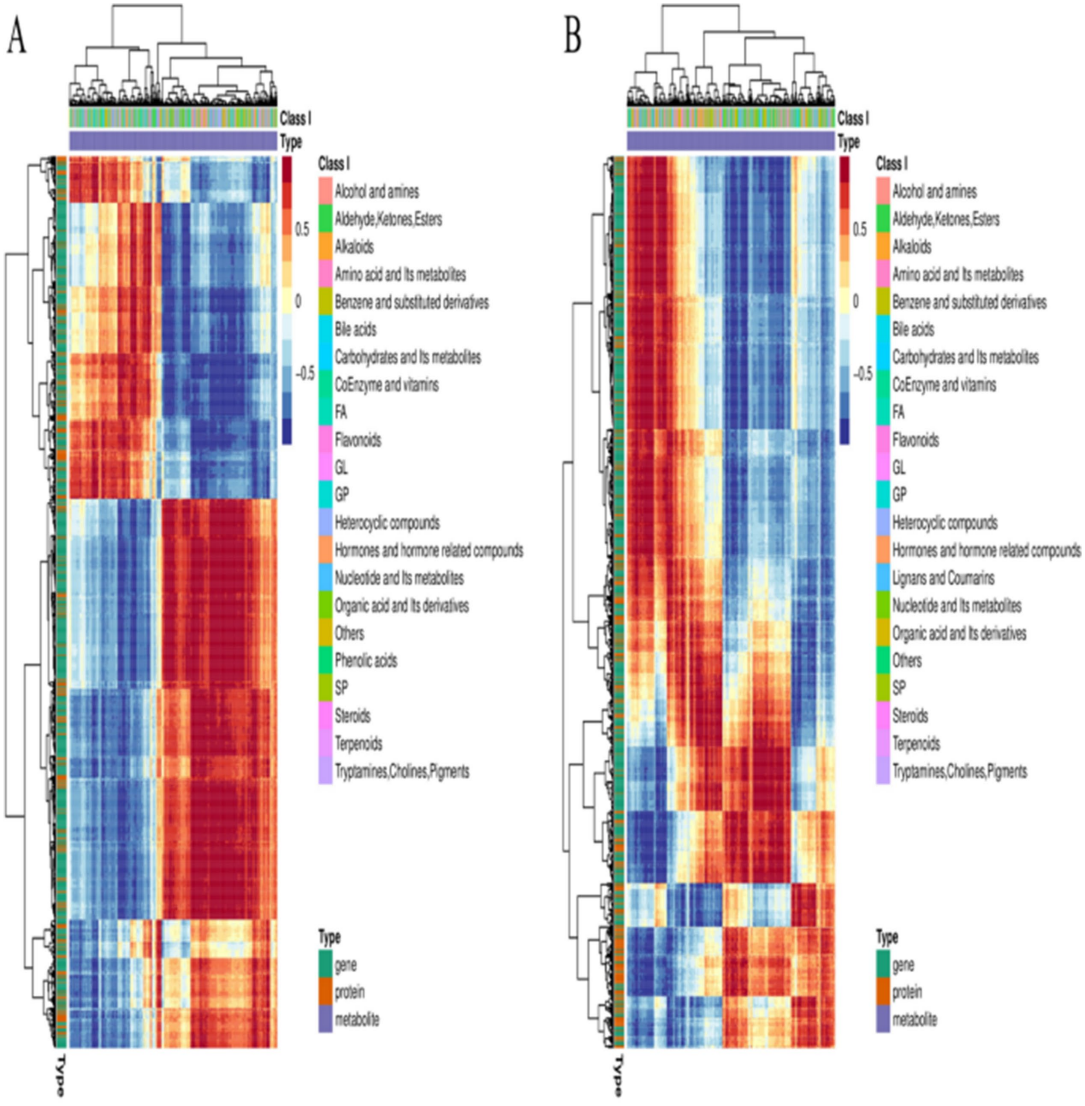
Combined heatmap of the results of the three omics analyses. **(A)** Significant DEGs, DEMs, and DEPs in *Pm*-YQ vs. *Pm*-E1. **(B)** Significant DEGs, DEMs, and DEPs in *Pm*-E1 vs. *Pm*-E2.

For better evaluation of the biological phenotypes and underlying regulatory mechanisms of the strains, the transcriptomic, metabolomic, and proteomic data were integrated. KEGG enrichment analysis revealed that, overall, the DEGs, DEMs, and DEPs were enriched in pathways involved in carbon and energy metabolism (Figure 7). Notably, the most significant enrichment was observed in pathways associated with amino sugar and nucleotide sugar metabolism, starch and sucrose metabolism, the pentose phosphate pathway, glycolysis/gluconeogenesis, the TCA cycle, and pyruvate metabolism. Furthermore, pathways associated with microbial adaptation, such as microbial metabolism in different environments, ABC transporters, and the two-component system, were also significantly enriched. The consistent enrichment patterns observed across all three omics layers indicate a coherent molecular response ranging from gene expression to metabolic flux. The heatmap (Figure 8) provides a visual summary of the differentially expressed molecules identified through the integrated analysis.

#### 3.5.2 Correlation analysis

**Figure 9:**
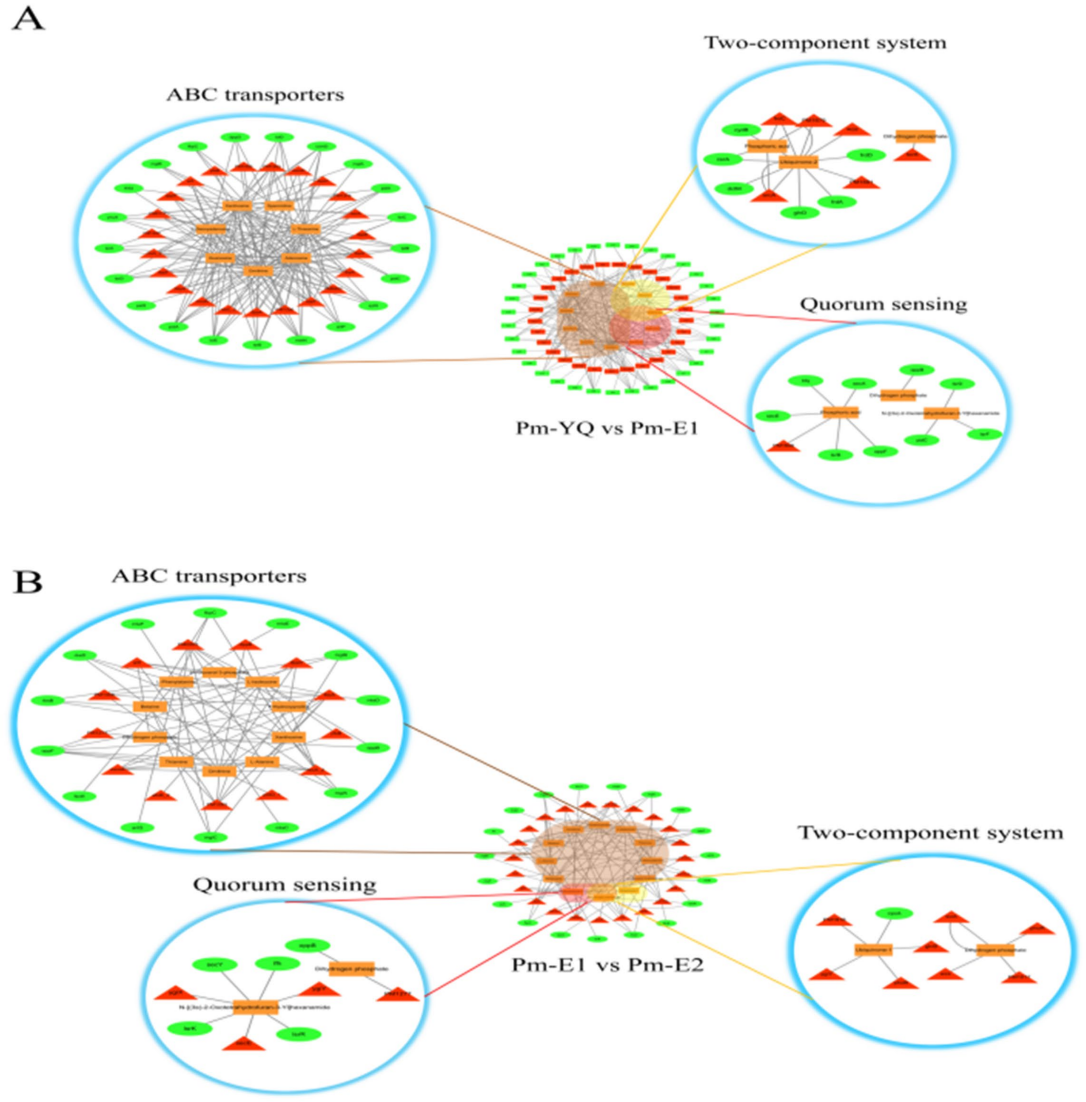
Transcriptomic, metabolomic, and proteomic association network. Circles represent genes, triangles indicate proteins, and squares represent metabolites. **(A)** Association network of *Pm*-YQ vs. *Pm*-E1. **(B)** Association network of *Pm*-E1 vs. *Pm*-E2.

The transcriptomic, proteomic, and metabolomic data were integrated, enabling the construction of multi-omics association networks focusing on three key pathways, namely, ABC transporters, the two-component system, and QS, with close associations with bacterial antibiotic resistance. The aim was to examine the mechanism underlying the regulatory activity of the QS signaling molecule C6 on resistance to ENR in *Pm*. As shown in Figure 9, although the multi-omics data from the two groups exhibited varying degrees of association across all three pathways, the most significant interaction network was observed for the ABC transporter pathway. This suggests that bacterial strains initially perceive antibiotic stress through the two-component system, leading to the regulation of the expression of ABC transporters to induce drug efflux, ultimately resulting in the development of resistance. Notably, following exogenous supplementation of the C6 QS signaling molecule, a significant increase was observed in the number of molecules associated with the ABC transporter pathway. This further suggested that the C6 signaling molecule may specifically modulate the expression of ABC transporters, thereby promoting drug efflux activity and ultimately enhancing bacterial resistance to ENR.

## 4 Discussion

Bovine respiratory diseases caused by *Pm* infection have had severe consequences for the recent development of the beef cattle industry in China, resulting in substantial economic losses. Fluoroquinolones are widely used as first-line antimicrobial agents in veterinary practice for treating *Pm* infections due to their potent bactericidal activity. However, driven by antibiotic misuse and natural selection, there has been a steady increase in *Pm* resistance to fluoroquinolone antibiotics, posing a serious threat to the beef cattle industry (Dhayalan et al., 2024). ENR, a fluoroquinolone antibacterial drug, interferes with the processes of DNA replication, transcription, and repair in bacteria by inhibiting the activity of DNA gyrase (topoisomerase II) and topoisomerase IV, ultimately resulting in DNA damage and death (Aboyadak and Ali, 2024; Xi, 2024). In this study, transcriptome sequencing revealed that treatment with sub-inhibitory concentrations of ENR led to marked down-regulation of genes involved in DNA synthesis, such as *nrdD*, consistent with the antibacterial mechanism of ENR (Torrents et al., 2000). Concurrently, the expression of genes involved in the bacterial SOS response, including *recA*, *recX*, and *LexA*, was significantly up-regulated. The SOS response is a critical mechanism associated with bacterial repair of DNA damage. Damage to bacterial DNA induces binding of the RecA protein to the exposed single-stranded DNA to form RecA-ssDNA nucleoprotein filaments, which mediate the autocleavage of the *lexA* repressor. This process derepresses the SOS response and initiates repair of the DNA damage (Mo et al., 2018; Cox, 2003; Xu et al., 2024; Cárdenas et al., 2012). The proteome sequencing showed significantly up-regulation expression of RecA, RecX, and other proteins, further verifying the activation of this pathway. Additionally, the expression of genes related to bacterial metabolism, including *mglB*, *glpT*, and *glpA*, was also significantly down-regulated, indicating an overall suppression of bacterial metabolic activities. The metabolome sequencing results showed general down-regulation of metabolic pathways involving various substances, corroborating the transcriptome sequencing findings. These results suggest that stress caused by sub-inhibitory concentrations of enrofloxacin induces the bacterial strain to reduce its metabolic level to conserve energy, thereby prioritizing energy allocation for the repair of DNA damage resulting from enrofloxacin exposure. In summary, we hypothesize that the bacterial strain enhances its resistance to ENR primarily through activation of the SOS response to initiate DNA damage repair, while simultaneously reducing energy consumption to allocate sufficient resources for DNA repair.

There have been numerous studies on the regulation of bacterial virulence, acid tolerance, and biofilm formation by Type I QS systems. However, to date, there has been limited investigation of their roles in the regulation of antibiotic resistance in bacteria, and almost nothing is known about their influence on resistance in *Pm*. Notably, the genome of *Pm* may lack typical LuxR-type receptors; however, studies have indicated that cells lacking LuxR homologs can still respond to AHL signaling molecules through alternative receptors. Therefore, the exogenous AHL signaling molecules supplemented in this study may not act via reliance on a complete canonical QS system, but are more likely attributable to the direct or indirect biological activities of the AHL molecules themselves (Delago et al., 2021). This study demonstrates that treatment of bacteria with the AHL signaling molecule C6 significantly modulated resistance to ENR. The results of the transcriptomic, metabolomic, and proteomic sequencing suggest that C6 may regulate bacterial resistance to ENR through three pathways.

First, C6 can modulate the bacterial SOS response. The SOS response system is known to play a critical role in the development of antibiotic resistance in bacteria. As a conserved regulatory network, it initiates appropriate responses to the presence of DNA damage, not only inducing DNA repair but also increasing the mutation frequency of target sites associated with drug resistance, thereby promoting the development of resistance (Ulrich et al., 2013; Kim et al., 2013). This study found that after the addition of sub-inhibitory concentrations of ENR, the expression of core SOS response genes, such as *recA* and *recX*, was significantly up-regulated. Furthermore, the subsequent introduction of the C6 signaling molecule led to a further up-regulation of these genes. Meanwhile, proteomic sequencing revealed significant up-regulation of proteins such as RecA and RecX, verifying the observed changes in gene expression. Additionally, the metabolomic sequencing results indicated significant down-regulation of multiple metabolic processes in the bacterial strain, resulting in reduced energy consumption. Based on these findings, it is therefore suggested that AHL signaling molecule C6 may positively regulate the expression of genes involved in DNA recombination repair such as *recA*, thereby activating and reinforcing the SOS repair pathway. In addition, the reduction in non-essential energy consumption provides sufficient energy for the bacterial SOS response, ultimately maintaining bacterial DNA integrity and enhancing bacterial resistance to ENR.

Second, C6 can impact the permeability of the bacterial outer membrane by activating the CpxAR two-component system. This system represents a regulatory hub involved in environmental adaptation found ubiquitously in Gram-negative bacteria, and is composed of the membrane-associated histidine kinase CpxA, the cytoplasmic response regulator CpxR, and the periplasmic accessory factor CpxP (Masi et al., 2020; Hunke et al., 2012). This system regulates various bacterial processes, including biofilm formation, environmental adaptation, antibiotic resistance, and virulence factor expression (Gahlot et al., 2022; Hirakawa et al., 2005; He et al., 2022). This study, through multi-omics sequencing, revealed that the addition of AHL signaling molecule C6 led to significant enrichment of genes, metabolites, and proteins associated with the two-component system in the bacterial strain, most notably up-regulation of the *cpxA* gene, indicating that AHL signaling molecule C6 may activate the CpxAR system either directly or indirectly. Additionally, significant differential expression was observed in outer membrane proteins and their associated regulatory genes, as seen in the marked down-regulation of the *ompA* gene. Studies have shown that the outer membrane protein OmpA not only participates in bacterium-host interactions, biofilm formation, and serum resistance but also represents an important channel enabling penetration of the antibiotic into the bacterium (Scribano et al., 2024). Furthermore, Hu et al. found that the CpxAR system can regulate *Salmonella Typhimurium* resistance to ceftriaxone by influencing the expression of the outer membrane proteins OmpD and STM1530 (Hu et al., 2011). Together, these findings suggest that the AHL signaling molecule C6 may enhance bacterial resistance to ENR by activating the CpxAR two-component system, which in turn modulates the expression of genes encoding outer membrane proteins, reduces the permeability of the outer membrane, and decreases the rate and total amount of antibiotic entry.

Third, C6 can contribute to the regulation of drug efflux mediated by ABC transporter systems. The ABC superfamily, one of the largest families of transmembrane proteins in bacteria, plays a significant role in the active efflux of antibiotics. When antibiotics penetrate the outer membrane and enter the cytoplasm, they may be recognized and bound directly by specific ABC transporters, utilizing energy derived from ATP hydrolysis to actively transport the drugs from the cell (Thomas and Tampé, 2020). The present study found that the addition of the C6 signaling molecule to bacteria led to significant up-regulation of multiple genes, metabolites, and proteins within the ABC transporter pathway, with notable increases in the expression of the *bacA* gene encoding the YddA transporter. YddA was initially proposed as a putative drug efflux transporter in *E. coli*. Subsequent research by Feng et al. confirmed that YddA is a multidrug efflux transporter belonging to the ABC family that relies on ATP hydrolysis for energy, and can recognize and transport a variety of structurally and functionally unrelated substrates, including quinolone drugs, and exhibits typical multidrug efflux characteristics. Moreover, that study observed markedly increased transcription of the YddA-associated gene when quinolone drug concentrations were raised (Feng et al., 2020). Combined with the results of this study, these findings suggest that the AHL signaling molecule C6 can enhance the active efflux of ENR by *Pm* by regulating the expression of the ABC family *YddA* drug efflux transporter, thereby mediating *Pm* resistance to ENR.

Based on multi-omics sequencing analysis, this study provides preliminary evidence that C6 may influence bacterial resistance to ENR through various pathways, including activation of the SOS response, modulation of the CpxAR two-component system mediating outer membrane permeability, and regulation of ABC transporter-mediated drug efflux. These findings not only elucidate the QS-mediated resistance mechanism in *Pm* but also provide a theoretical basis and suggest potential targets for the development of novel QSIs. However, it should be noted that the integrated multi-omics approach employed in this study is fundamentally correlative in nature, and its conclusions are subject to certain limitations. Multi-omics techniques typically provide static molecular snapshots at specific time points, making it difficult to fully capture the inherent dynamic complexity involved in temporal changes in QS regulation. The interpretation of the data also relied on the completeness and accuracy of existing annotation databases, which may have affected the reliability of the pathway inferences. Furthermore, while multi-omics data can effectively delineate global molecular trends and generate important hypotheses, they cannot provide direct confirmation of specific regulatory mechanisms. For example, the multiple drug resistance mechanisms hypothesized in this study still require functional validation through targeted follow-up experiments such as gene knockout, complementation assays, or protein function interference. Future studies should investigate the specific molecular mechanisms by which C6 regulates drug resistance in *Pm* to provide a theoretical foundation for the development of new resistance inhibitors and antimicrobial agents, as well as opening new avenues for the prevention and treatment of bovine pasteurellosis.

## 5 Conclusion

This study found that the addition of 200 μM (1 × 10^−3^ mol/L) AHL signaling molecule C6 to the test strains significantly enhanced their resistance to ENR. Based on multi-omics analyses, it is hypothesized that the AHL signaling molecule may regulate the resistance of the test strains to ENR through three potential pathways: First, it modulates the bacterial SOS response by upregulating the expression of DNA recombination and repair genes such as *recA*, thereby activating and enhancing the SOS repair pathway to protect bacterial DNA integrity and consequently strengthen bacterial resistance to ENR. Second, it activates the CpxAR two-component system to modulate outer membrane permeability, reducing both the rate and total amount of antibiotic entry into bacterial cells, thus increasing bacterial resistance to ENR. Third, it is involved in regulating the ABC transporter-mediated drug efflux system by modulating the expression of the ABC family efflux transporter *YddA*, enhancing the active efflux capacity of *Pm* against ENR, thereby mediating *Pm* resistance to ENR.

## Data Availability

All sequences have been deposited in the NCBI Sequence Read Archive, the MetaboLights and the ProteomeXchange database under accession numbers PRJNA1343251, PXD069645 and MTBLS13502, respectively, and are publicly accessible via the following links: https://www.ncbi.nlm.nih.gov/bioproject/PRJNA1343251, https://proteomecentral.proteomexchange.org/cgi/GetDataset?ID=PXD069645 and https://www.ebi.ac.uk/metabolights/editor/MTBLS13502/descriptors.

## Footnotes

1
http://www.r-project.org/
