# Supplementary figures and images for: Regulation of drug resistance to enrofloxacin in Pasteurella multocida strains from cattle by quorum-sensing acyl-homoserine lactone signaling molecules
Source: Front Microbiol. 2026 Jan 26;17:1766173. doi: 10.3389/fmicb.2026.1766173 (PMC12883784; doi:10.3389/fmicb.2026.1766173)

## Slide 1
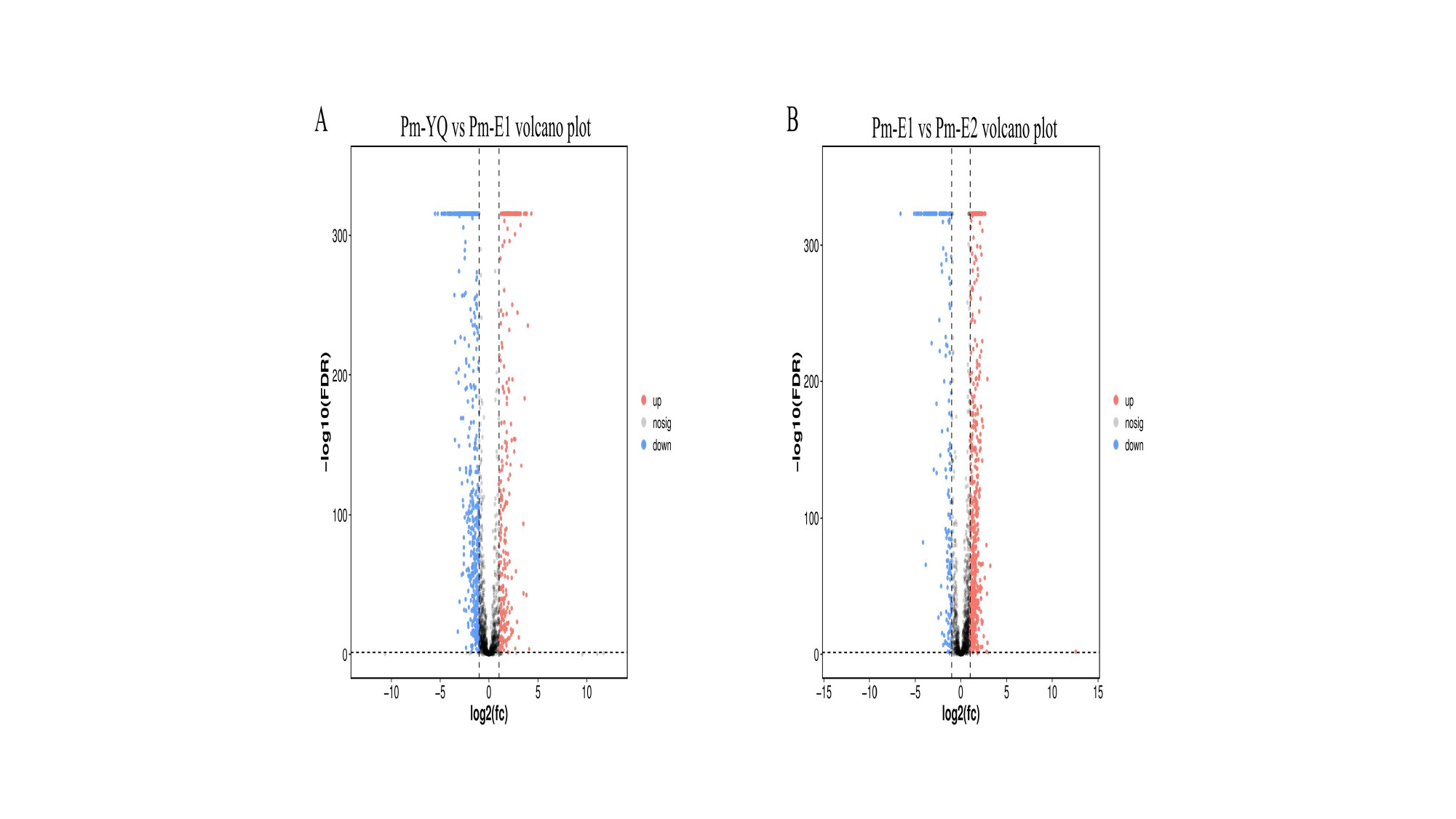

## Slide 2
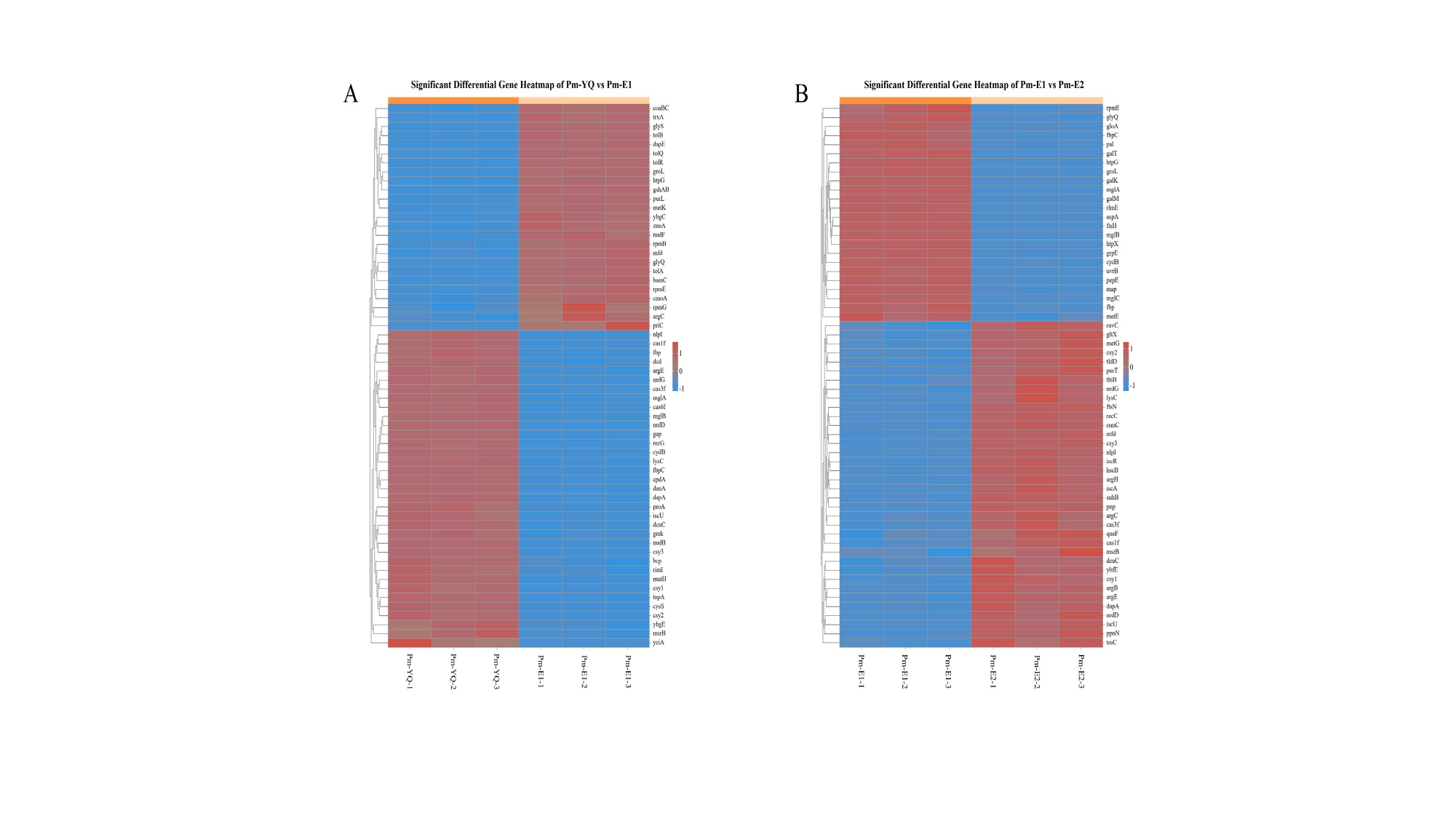

## Slide 3
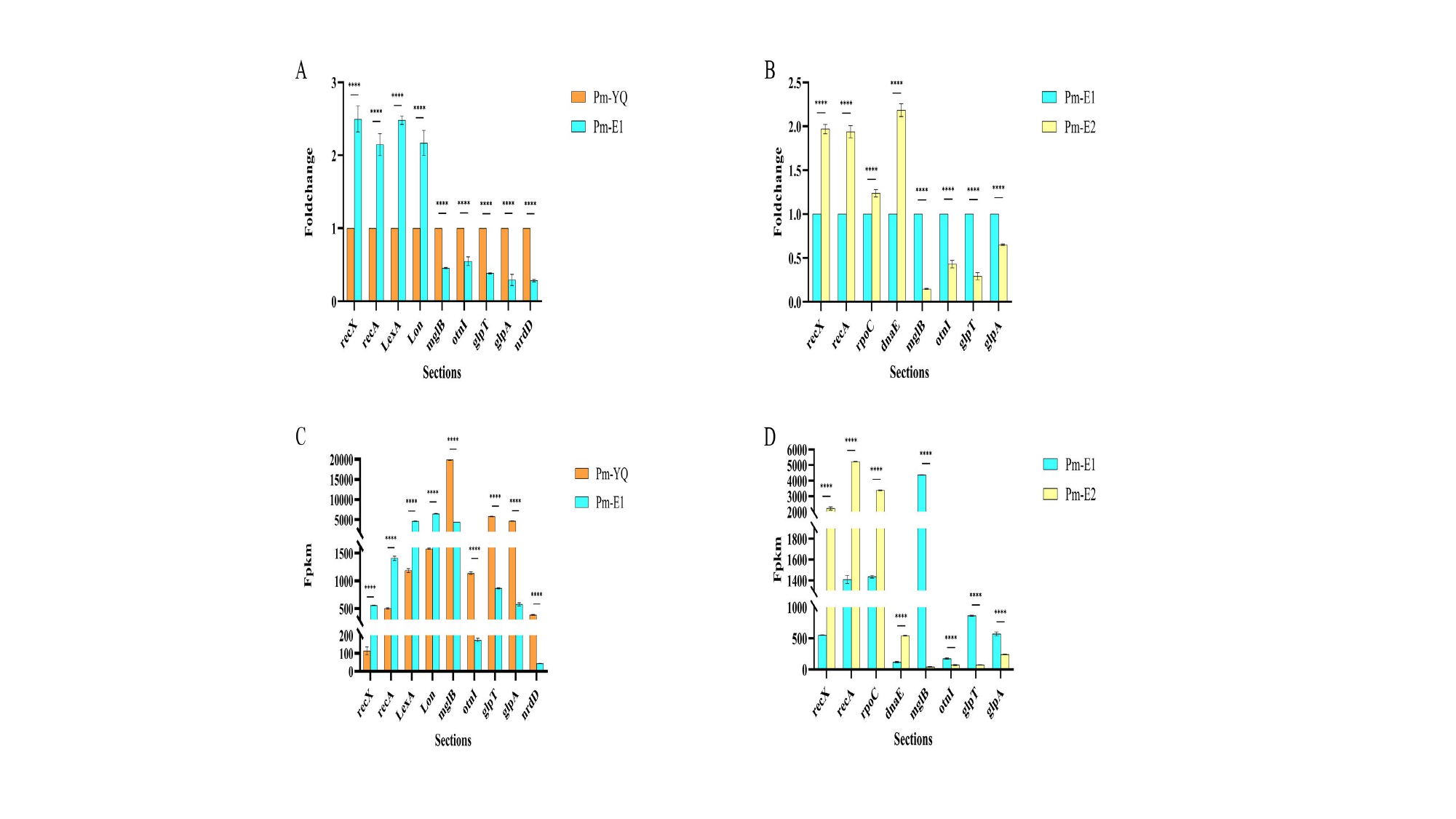

## Slide 4
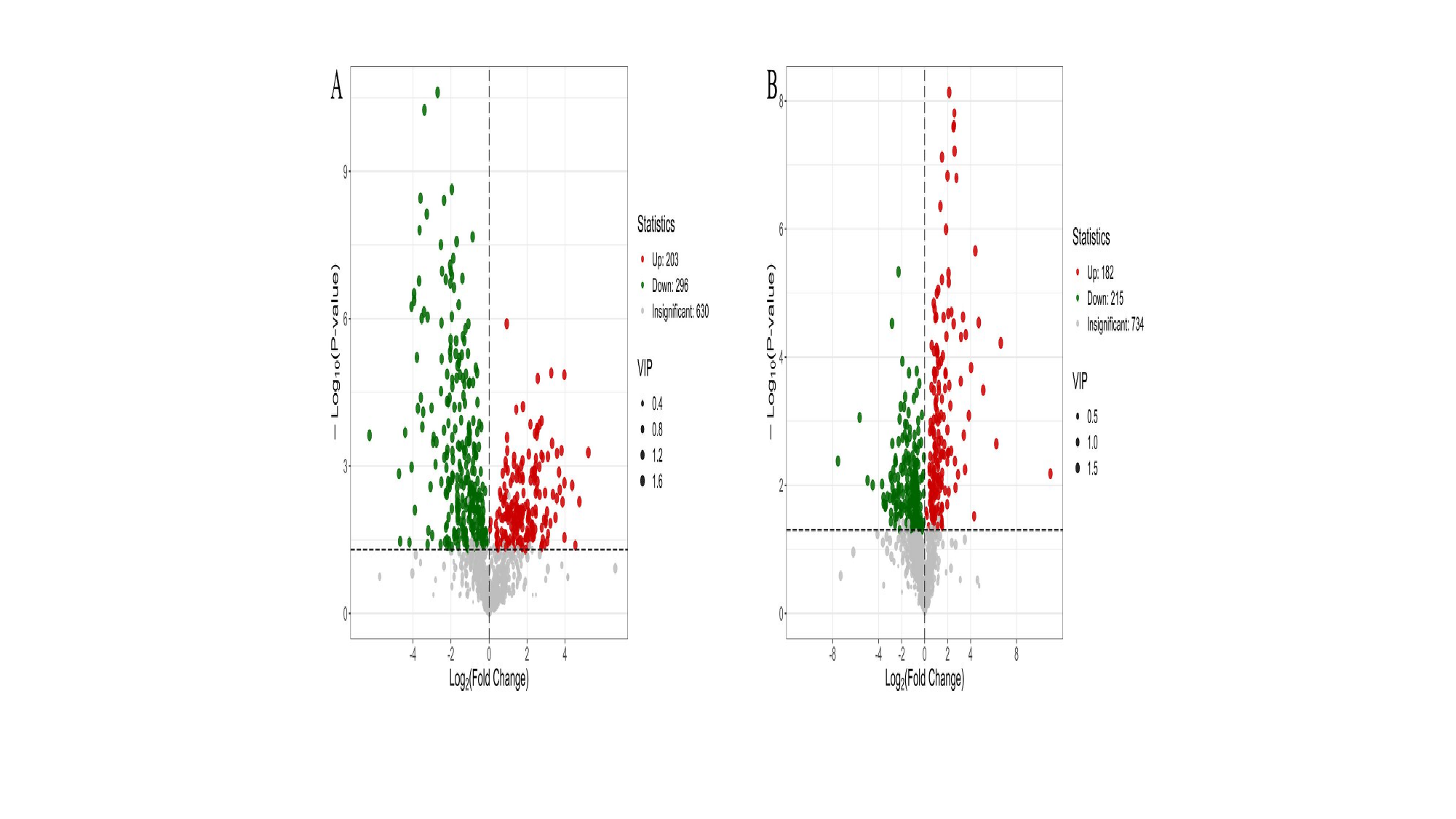

## Slide 5
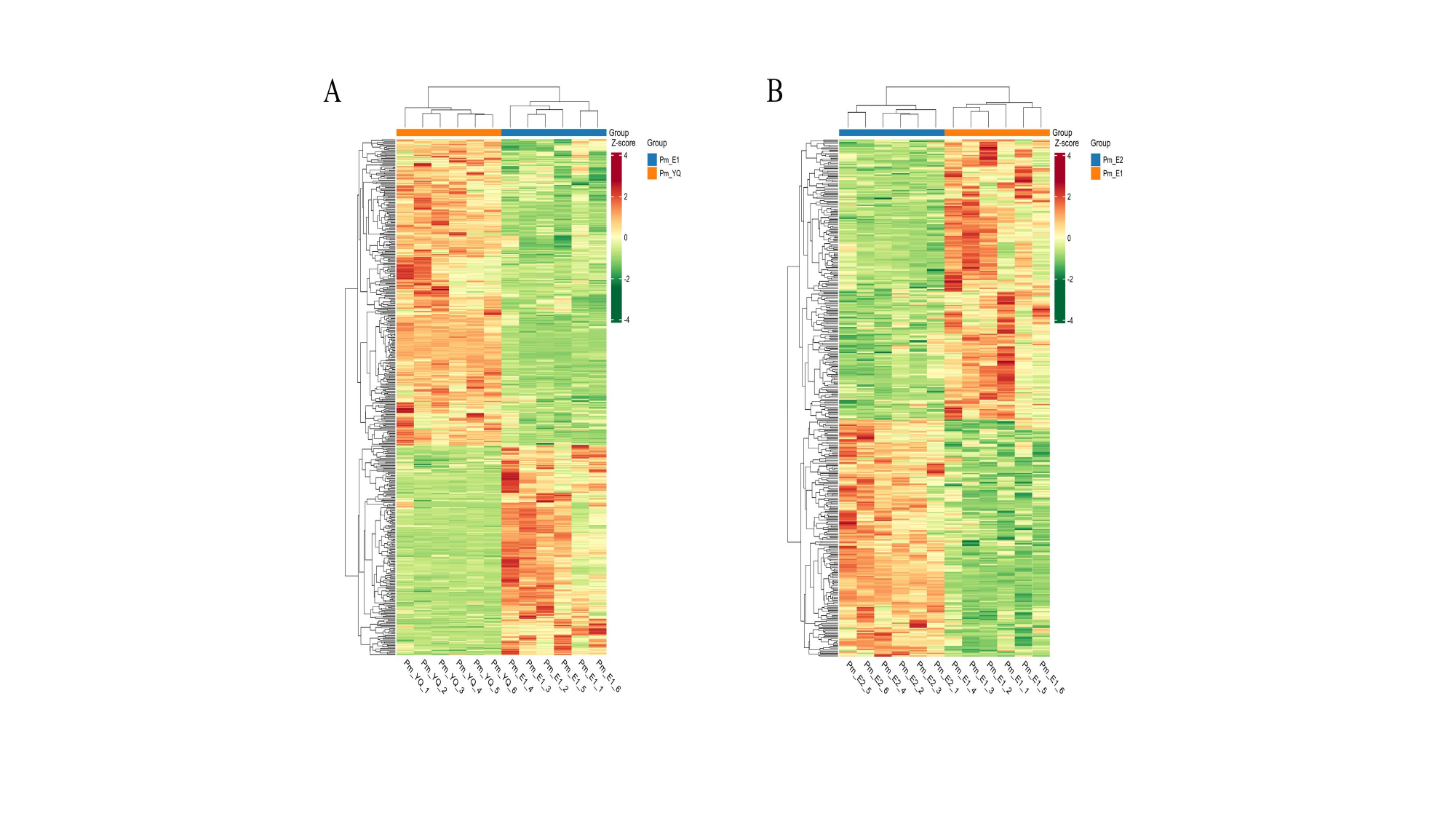

## Slide 6
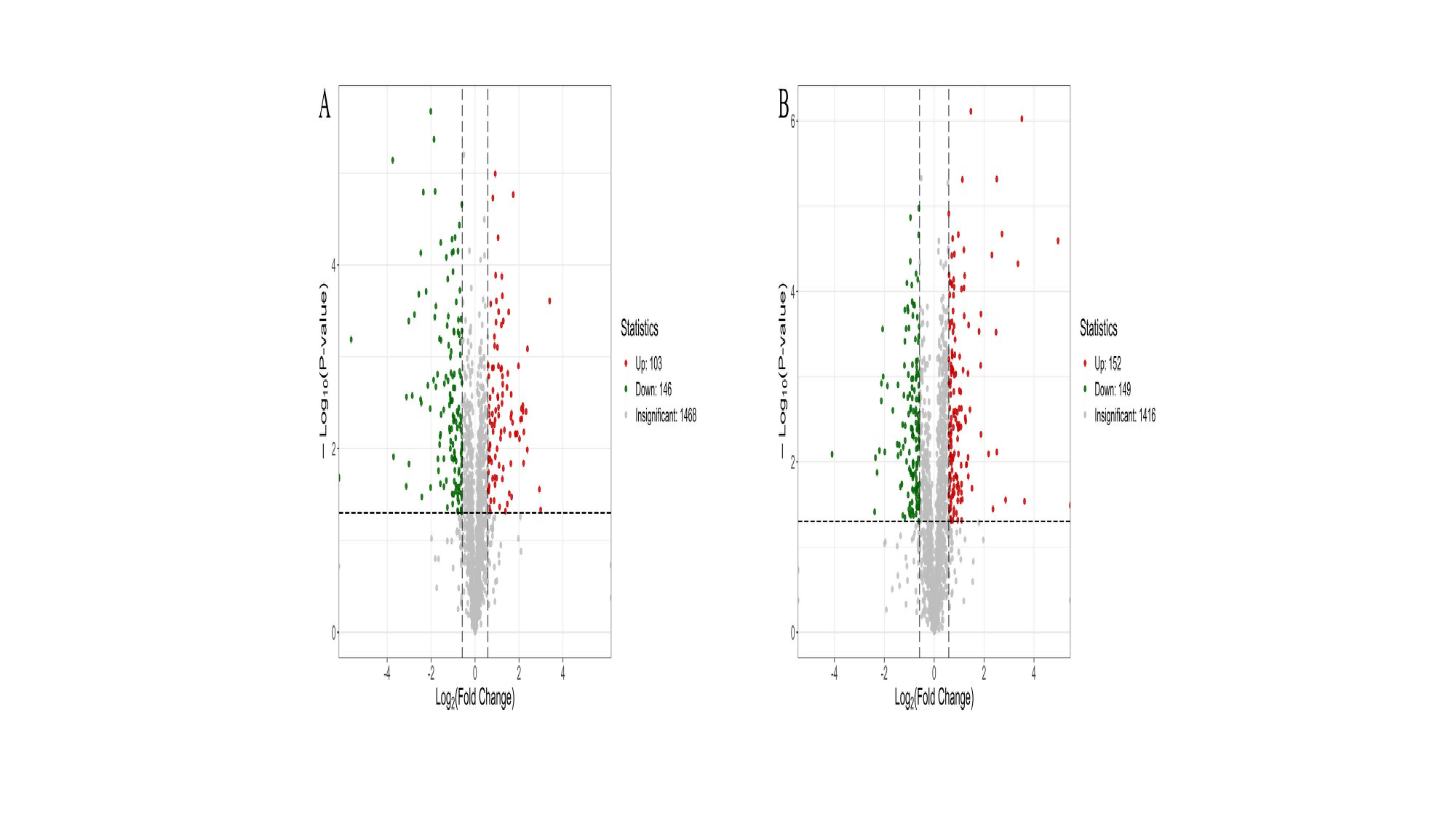

## Slide 7
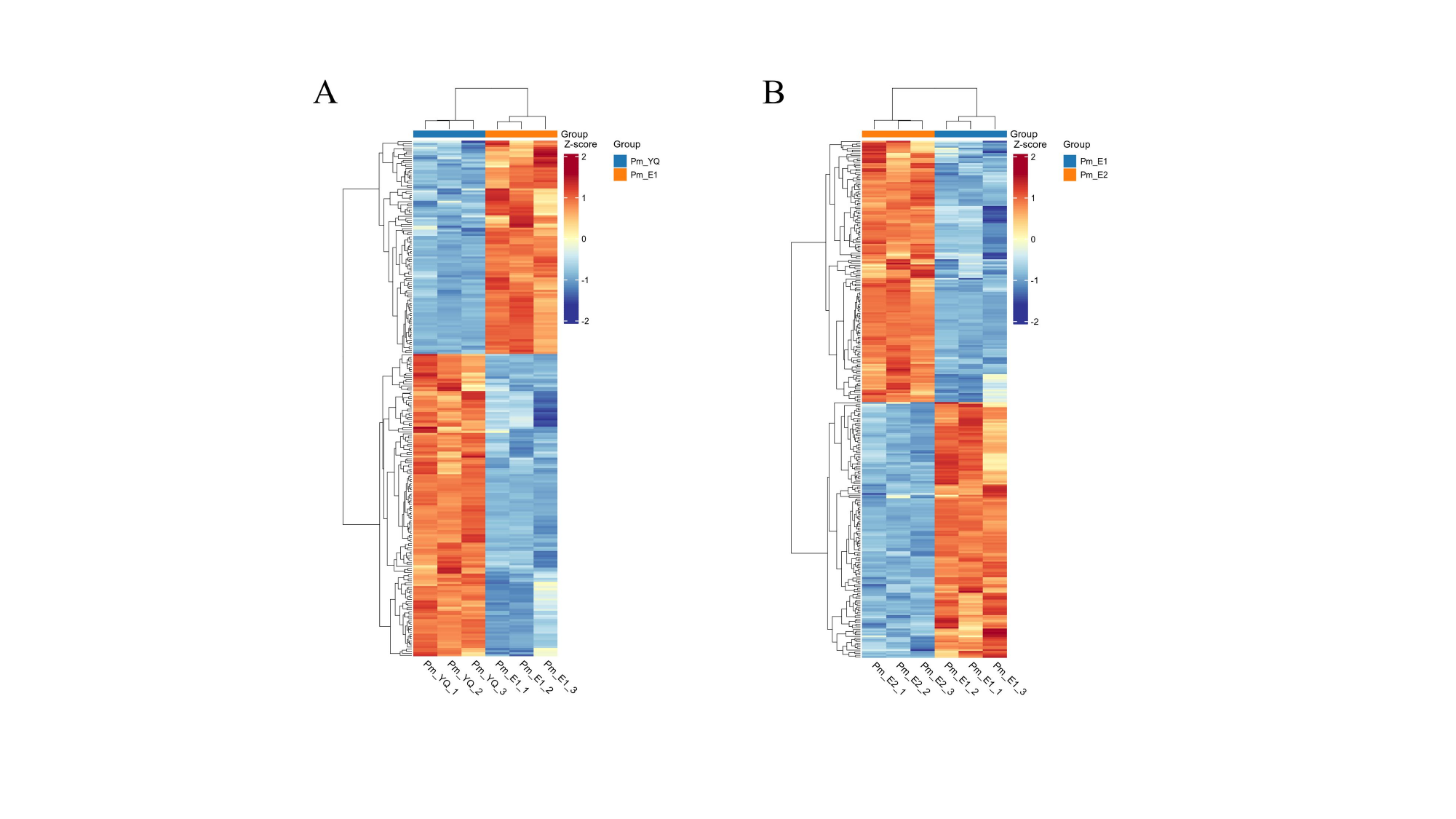

Supplement: Supplementary file 1 [file Supplementary_file_1.pptx]
