# Supplementary material for: Regulation of drug resistance to enrofloxacin in Pasteurella multocida strains from cattle by quorum-sensing acyl-homoserine lactone signaling molecules
Source: Front Microbiol. 2026 Jan 26;17:1766173. doi: 10.3389/fmicb.2026.1766173 (PMC12883784; doi:10.3389/fmicb.2026.1766173)
Supplement: Supplementary file 2 [file Table_1.docx]

Table S1 Validation of gene primer sequences

| Primer name | Primer sequence (5´→3´) |
| --- | --- |
| recX-F | CCACAGTATGCCGAAATCA |
| recX-R | AATGTGCAAATTGGTCAGG |
| recA-F | GATGCGTTAGTGCGTTCAG |
| recA-R | GTCACCCATTTCTCCTTCA |
| LexA-F | TACAGGTATGCCGCCAACA |
| LexA-R | CTCAATCACGCCTTTACGC |
| Lon-F | AAAACCGTGTTGGTGAAGT |
| Lon-R | CCCTTACCCAAGACTGACG |
| mglB-F | CGCTAACAACGATGGTATG |
| mglB-R | TAATTGGAGGACTTCTGGT |
| otnI-F | GTTGATCGGGATAATGTCT |
| otnI-R | ATGTGCCACTTTGTCTTTC |
| glpT-F | TAATCCGCTACGGTGTCTT |
| glpT-R | AATAGTGTACGCCCAAGCA |
| glpA-F | CGCTGGCGAAGTGCGTTAT |
| glpA-R | ACTCCGCCTGACAAGTCCC |
| nrdD-F | ACTATGGGACCAATACACC |
| nrdD-R | ACCACATTTCGGACAAGTA |
| q16s-F | GAGGCGACCTTGAAATACC |
| q16s-R | CCAGTCAAACTACCCACCA |
